# Comparative Proteomics of Three Species of Ammonia-Oxidizing Bacteria

**DOI:** 10.3389/fmicb.2018.00938

**Published:** 2018-05-14

**Authors:** Jackie K. Zorz, Jessica A. Kozlowski, Lisa Y. Stein, Marc Strous, Manuel Kleiner

**Affiliations:** ^1^Department of Geoscience, University of Calgary, Calgary, AB, Canada; ^2^Department of Ecogenomics and Systems Biology, Division Archaea Biology and Ecogenomics, University of Vienna, Vienna, Austria; ^3^Department of Biological Sciences, University of Alberta, Edmonton, AB, Canada; ^4^Department of Plant and Microbial Biology, North Carolina State University, Raleigh, NC, United States

**Keywords:** nitrification, *Nitrosomonas europaea*, *Nitrosomonas ureae*, *Nitrosospira multiformis*, Calvin-Benson-Bassham cycle, Q Exactive Plus, proteomics, ammonia-oxidizing bacteria (AOB)

## Abstract

Ammonia-oxidizing bacteria (AOB) are important members of terrestrial, marine, and industrial microbial communities and play a fundamental role in the Nitrogen cycle within these systems. They are responsible for the first step of nitrification, ammonia oxidation to nitrite. Although AOB are widespread and essential to environmental and industrial systems, where they regularly experience fluctuations in ammonia availability, no comparative studies of the physiological response of diverse AOB species at the protein level exist. In the present study, we used 1D-LC-MS/MS proteomics to compare the metabolism and physiology of three species of ammonia AOB, *Nitrosomonas europaea*, *Nitrosospira multiformis*, and *Nitrosomonas ureae*, under ammonia replete and ammonia starved conditions. Additionally, we compared the expression of orthologous genes to determine the major differences in the proteome composition of the three species. We found that approximately one-third of the predicted proteome was expressed in each species and that proteins for the key metabolic processes, ammonia oxidation and carbon fixation, were among the most abundant. The red copper protein, nitrosocyanin was highly abundant in all three species hinting toward its possible role as a central metabolic enzyme in AOB. The proteomic data also allowed us to identify pyrophosphate-dependent 6-phosphofructokinase as the potential enzyme replacing the Calvin-Benson-Bassham cycle enzyme Fructose-1,6-bisphosphatase missing in *N. multiformis* and *N. ureae*. Additionally, between species, there were statistically significant differences in the expression of many abundant proteins, including those related to nitrogen metabolism (nitrite reductase), motility (flagellin), cell growth and division (FtsH), and stress response (rubrerythrin). The three species did not exhibit a starvation response at the proteome level after 24 h of ammonia starvation, however, the levels of the RuBisCO enzyme were consistently reduced after the starvation period, suggesting a decrease in capacity for biomass accumulation. This study presents the first published proteomes of *N. ureae* and *N. multiformis*, and the first comparative proteomics study of ammonia-oxidizing bacteria, which gives new insights into consistent metabolic features and differences between members of this environmentally and industrially important group.

## Introduction

Nitrification is an essential process of the nitrogen cycle in terrestrial, aquatic, and wastewater systems that links reduced and oxidized pools of inorganic nitrogen (Gruber and Galloway, 2008). Nitrification is classically considered a two-step process with the first and rate limiting step, ammonia (NH_3_) oxidation to nitrite (NO_2_^-^), performed by ammonia-oxidizing bacteria (AOB) and archaea, and NO_2_^-^ oxidation to nitrate (NO_3_^-^) performed by nitrite-oxidizing microorganisms (Kowalchuk and Stephen, 2001; Klotz and Stein, 2011). Recently organisms have been discovered that can perform the complete process of NH_3_ oxidation to NO_3_^-^ (Daims et al., 2015; Van Kessel et al., 2015). This study focuses solely on the AOB. Ammonia-oxidation by AOB is catalyzed aerobically via two characterized enzymes, ammonia monooxygenase (AMO) which oxidizes NH_3_ to hydroxylamine (NH_2_OH), and hydroxylamine dehydrogenase (HAO) which oxidizes NH_2_OH most likely to nitric oxide (NO) (Caranto and Lancaster, 2017). A third enzyme that oxidizes NO to nitrite (NO_2_^-^) has not yet been characterized and was only recently proposed as a third requisite enzyme in the ammonia-oxidation pathway.

The AOB are generally obligate chemolithotrophs living solely off the energy from oxidizing NH_3_ with oxygen as the terminal electron acceptor. They are autotrophic and obtain carbon through fixation of carbon dioxide using the Calvin-Benson-Bassham (CBB) cycle (Schramm et al., 1998; Utåker et al., 2002), though the capacity for mixotrophy has been observed in *Nitrosomonas europaea* (Hommes et al., 2003). AOB are also capable of reducing NO_2_^-^ to N_2_O via NO through a process termed nitrifier denitrification, which enables maintenance of intracellular redox balance under conditions of oxygen limitation (Stein, 2011).

Niche differentiation among strains of AOB and AOA has been attributed to differences in affinities for NH_3_, temperature, and pH, among other factors (Bollmann and Laanbroek, 2002; Nicol et al., 2008; Fierer et al., 2009; Martens-Habbena et al., 2009). Differences in NH_3_ affinities in particular dictate oligotrophic versus eutrophic environmental preferences within distinct groups of AOB (Klotz and Stein, 2011). The three species investigated in this study, *N. europaea* ATCC 19718, *Nitrosospira multiformis* ATCC 25196, and *Nitrosomonas ureae* Nm10 are all ecologically relevant strains of AOB and have complete genome sequences available (Chain et al., 2003; Norton et al., 2008; Kozlowski et al., 2016b). They are phylogenetically diverse representing different clusters and ecotypes within betaproteobacterial AOB. *N. europaea* is a representative of cluster 7, *N. ureae* is a representative of cluster 6a, and *N. multiformis* is a representative of *Nitrosospira* cluster 3 (Purkhold et al., 2000; Kozlowski et al., 2016a). These strains are generally widespread, however, *N. europaea* is more often recovered from nitrogen-rich environments like wastewater treatment plants, whereas *Nitrosospira* strains are more often found in terrestrial or agricultural soil systems (Prosser et al., 2014). Organisms from *Nitrosomonas* cluster 6a (*N. ureae*) are often isolated from freshwater or marine environments (Speksnijder et al., 1998; Bollmann and Laanbroek, 2001) and are thus considered comparatively oligotrophic (Prosser et al., 2014).

The AOB in both environmental and industrial settings experience fluctuations in nutrient and substrate availability (Geets et al., 2006). Previous work has aimed to identify the starvation response of specific species of AOB by observing growth and enzyme activity (Sayavedra-Soto et al., 1996; Bollmann and Laanbroek, 2002), mRNA levels (Wei et al., 2004, 2006; Stein et al., 2013; Pérez et al., 2014; Mellbye et al., 2016), and more recently proteomes (Pellitteri-Hahn et al., 2011; Jiang et al., 2015; Sedlacek et al., 2016; Yu et al., 2018). However, past work has mainly been conducted on single species (primarily *N. europaea*) and in most cases, has focused on the response of genes related to nitrogen cycling, rather than whole-genome expression at the proteome level. This study uses proteomics to compare the diversity in gene expression across phylogenetically coherent and functionally similar bacteria under the same growth conditions: ammonia replete and ammonia starved. Because each of the three AOB species is adapted to its own range of physicochemical parameters, the variance in protein expression will clearly show the range of genomic variability and flexibility. The results observed in this paper provide evidence about how genomic and proteomic expression governs niche preferences by individual species.

## Materials and Methods

### Culture Growth and Starvation

The three species of AOB, *N. europaea* ATCC 19718, *N. multiformis* ATCC 25196, and *N. ureae* Nm10, were grown at 22°C, in triplicate 300 ml cultures with 100 rpm of shaking, in the dark. We used 5 mM (NH_4_)_2_SO_4_ HEPES-buffered HK medium (0.2 mM MgSO_4_.7H_2_O, 1.0 mM CaCl_2_.2H_2_O, 1.0 mM KCl, 0.02% Phenol red, 15 mM HEPES buffer, trace solution, pH 7.8) (Krummel and Harms, 1982). Each 300 ml culture was inoculated with 6 ml of a 4 × 10^7^ to 5 × 10^7^ cells ml^-1^ culture, resulting in an initial density of approximately 1 × 10^6^ cells ml^-1^. We determined cell density daily using OD600 measurements (Supplementary Figure 1), and we converted OD600 values to cell density (cells ml^-1^) using standard curves of cell counts, which we generated by comparing cell counts with a Neubauer counting chamber to OD600 values. The pH of the cultures was maintained using sterile 10% sodium bicarbonate. Once cell density had reached late exponential phase and at least 1 × 10^7^ cells ml^-1^ (Supplementary Figure 1), we spun down the cultures at 15,000 × *g* for 15 min. Cells were resuspended and washed in 200 ml of ammonia-free HK media and spun down again (15,000 × *g* for 15 min). The pellet was then resuspended in 10 ml of ammonia-free HK media. We split each replicate, adding 5 ml of the cell suspension to 100 ml HK media with ammonia to act as a control and the remaining 5–100 ml HK media without ammonia for the starved treatment. This resulted in a total of six cultures per species. One of the starved cultures of *N. ureae* was lost during downstream sample processing. Both control and starved cultures were grown for 24 h at 100 rpm in the dark. After 24 h, we centrifuged the cultures (4,816 × *g* for 20 min) in a Sorvall ST40R centrifuge with a swinging bucket rotor (75003608) (Thermo Fisher Scientific), followed by centrifugation at 21,000 × *g* for 5 min in a microcentrifuge. Pellets were immediately stored at -80°C. Previous studies (e.g., Wei et al., 2006), demonstrated that less than 24 h of substrate starvation resulted in a significant starvation response in the transcriptome of *N. europaea*, so 24 h was chosen as the starvation period for the present study.

Ammonia and nitrite concentrations of media in the pre-culture prior to starvation, in starved cultures, and in control cultures were measured using standard colorimetric assays (Supplementary Table 1). Ammonia was measured using the assay from Sims et al. (1995), and nitrite was measured using a Griess test (Griess-Romijn van Eck, 1966).

### Protein Extraction

Protein was extracted using the filter-aided sample preparation (FASP) protocol as previously described (Wisniewski et al., 2009). Briefly, for lysis, we added SDT-lysis buffer [4% (w/v) SDS, 100 mM Tris–HCl, 0.1 M DTT] in a 1:10 pellet:buffer ratio to sample pellets. We heated samples at 95°C for 10 min with periodic vortexing. This was followed by 5 min of sonication in a Branson 8800 sonication water bath (Branson). Once lysed, we centrifuged samples for 5 min at 21,000 × *g* to clear debris. After this we followed the FASP protocol for the remaining steps. Approximate peptide concentrations were determined using a Micro BCA Protein Assay Kit (Thermo Fisher Scientific).

### Proteomics

Samples were analyzed by one-dimensional Liquid Chromatography with Tandem Mass Spectrometry (LC-MS/MS) on a Q Exactive Plus Hybrid Quadrupole-Orbitrap Mass Spectrometer (Thermo Fisher Scientific). The LC-MS/MS analysis was done as previously described (Kleiner et al., 2017). For each run, ∼1200 ng of peptide was loaded onto a 5 mm, 300 μm ID C18 Acclaim PepMap100 pre-column (Thermo Fisher Scientific) using an UltiMate^TM^ 3000 RSLCnano Liquid Chromatograph (Thermo Fisher Scientific). Peptides were then separated on a 50 cm × 75 μm analytical EASY-Spray column packed with PepMap RSLC C18, 2-μm material (Thermo Fisher Scientific) using a 260-min gradient as described in Kleiner et al. (2017). The column was heated to 45°C via an integrated heating module. The analytical column was connected via an EASY-Spray source to the Orbitrap Mass Spectrometer. In between each sample, two washes with acetonitrile and one blank were run to reduce and assess carry over. Eluting peptides were analyzed in the Orbitrap Mass Spectrometer as described by Petersen et al. (2016). Roughly 140,000 MS/MS spectra were acquired per sample run (Supplementary Table 1).

### Proteomics Analysis

A protein sequence database for each species was made using the reference proteomes from Uniprot of *N. europaea* (AL954747) (Chain et al., 2003), *N. multiformis* (CP000103) (Norton et al., 2008), and *N. ureae* (FNLN01000000) (Kozlowski et al., 2016b). The sequences of common contaminating proteins were also added to each database^1^. We used the program Proteome Discoverer version 2.0.0.802 (Thermo Fisher Scientific) to analyze the proteome data for each species separately. The MS/MS spectra for each species were searched against the protein sequence database using the Sequest HT node in Proteome Discoverer as described by Petersen et al. (2016). False discovery rates (FDRs) for peptide spectral matches (PSMs) were calculated and filtered using the Percolator Node in Proteome Discoverer (Spivak et al., 2009), using an FDR of 5% as the cut-off. Protein level FDRs were determined using FidoCT and filtered at 5% as well.

Search results for all samples were combined into a multiconsensus report with Proteome Discoverer. Relative protein abundances were calculated based on spectral counts that were normalized for protein length and the total number of spectra using the normalized spectral abundance factor (NSAF) approach (Zybailov et al., 2006). Perseus version 1.5.6.0 (Tyanova et al., 2016) and R (R Development Core Team, 2015) version 3.2.4 were used for the statistical analysis and visualization of the data. Prior to any statistical testing, we added a constant of 10^-10^ to all NSAF values and then applied a centered log-ratio (clr) transform (Aitchison, 1982; Fernandes et al., 2014) using the *clr* function from the *chemometrics* package in R (Kumar et al., 2014). For statistical tests, we also removed very low abundance proteins that did not have at least 20 spectral counts in total across all samples. We used *t*-tests to determine proteins that were statistically different between control and starved groups for each of the strains (*p* <0.05). In Perseus, to correct for multiple testing, we used permutation based calculation of FDR (Tusher et al., 2001). Average values reported in this study were calculated based on the triplicate control cultures.

### Analysis of Orthologs

We identified orthologs shared between the three strains of AOB using the program OrthoVenn (Wang et al., 2015) with the protein sequence files obtained from Uniprot as input. OrthoVenn provided tab-delimited tables of protein sequence accession numbers that indicated which protein sequences represented orthologs. We merged the ortholog tables with the protein quantification tables using the VLOOKUP function in Excel. We used pairwise *t*-tests (*p* < 0.05) to identify statistically significant differences in the expression of orthologous proteins between strains. We corrected for multiple testing using permutation-based FDR implemented in the program Perseus version 1.5.6.0 (Tyanova et al., 2016). The NSAF values were transformed using the clr transformation prior to testing as described above.

## Results and Discussion

### Major Protein Expression Patterns in AOB Proteomes Under Ammonia Replete Conditions

A total of 891, 1064, and 814 proteins were identified from *N. ureae*, *N. multiformis*, and *N. europaea*, representing approximately 30, 37, and 32% of the predicted proteome for each species, respectively. Of these, 181 (20% of expressed proteins), 111 (10% of expressed proteins), and 107 (13% of expressed proteins) were listed as uncharacterized proteins for *N. ureae*, *N. multiformis*, and *N. europaea*, respectively.

A previous proteomics study on *N. europaea* found a total of 876 expressed proteins (34% of predicted proteome) (Pellitteri-Hahn et al., 2011), which is remarkably similar to the 814 expressed proteins (32% of predicted proteome) identified in this present study. In comparison, proteome analysis of *Nitrosomonas eutropha*, an eutrophic species, found 24% of the predicted proteome expressed (Wessels et al., 2011). In oligotrophic AOA species, separate proteome analyses revealed that *Nitrososphaera viennensis* expressed 1503 proteins, representing 48% of its genome (Kerou et al., 2016), while *Nitrosopelagicus brevis* which has a very streamlined genome, expressed 70% of its predicted proteome (Santoro et al., 2015).

There were some major differences in the most highly expressed proteins across the species (**Table 1**). In all three strains, AMO subunit B was either the most highly expressed (*N. ureae*, *N. multiformis*), or the second most highly expressed (*N. europaea*) protein. The most abundant protein in *N. europaea* was a general diffusion Gram-negative porin (Q82S02), which accounted for 4.36% of the strain’s normalized proteome. Orthologous porin proteins in *N. ureae* (A0A0S3AG80) and *N. multiformis* (Q2Y5X1) were also relatively highly expressed (**Table 1**). In the previous proteomics study conducted on *N. europaea*, the same general diffusion Gram-negative porin was identified as the most abundant protein (Pellitteri-Hahn et al., 2011). This confirms that there is some continuity between quantitative proteomics studies, even when using varying proteomics techniques and instruments. The second most abundant protein in *N. ureae* was nitrite reductase, but this enzyme was not highly expressed in the other two AOB (see following sections). Other highly expressed proteins included chaperonin proteins, which assist protein folding, elongation factor Tu, involved in protein translation, nitrosocyanin (NcyA), and rubrerythrin. In general, these highly expressed proteins play a role in key metabolic pathways, as well as basic cell function and maintenance.

**Table 1:** Most highly expressed proteins from *N. ureae*, *N. europaea*, and *N. multiformis* under ammonia replete conditions.

Rank	*N. ureae* Protein	Nu NSAF^∗^	*N. europaea* Protein	Ne NSAF^∗^	*N. multiformis* Protein	Nm NSAF^∗^
1	Ammonia monooxygenase subunit B (A0A0S3AFP0)	3.49	General diffusion Gram-negative porin (Q82S02)	4.36	Ammonia monooxygenase subunit B (Q2Y6K6)	3.81
2	Nitrite reductase (A0A0S3AFD2)	2.89	Ammonia monooxygenase subunit B (Q04508)	3.46	RubisCO small subunit (Q2YB79)	2.38
3	Nitrosocyanin (A0A0S3AFA2)	2.27	Uncharacterized Protein (Q82TI0)	2.16	Nitrosocyanin (Q2Y8L9)	1.80
4	General diffusion Gram-negative porin (A0A0S3AG80)	1.98	Peptidoglycan-binding protein (Q82XN8)	1.79	RubisCO large chain (Q2YB78)	1.79
5	60 kDa chaperonin (A0A0S3AFK1)	1.87	Bacterial histone-like DNA-binding protein (Q82SU7)	1.77	10 kDa chaperonin (Q2Y6I5)	1.57
6	10 kDa chaperonin (A0A0S3AG00)	1.86	60 kDa chaperonin (Q82Y60)	1.69	60 kDa chaperonin (Q2Y6I6)	1.39
7	Uncharacterized protein (A0A0S3AIB4)	1.55	Nitrosocyanin (Q820S6)	1.69	Bacterial outer membrane protein (Q2Y6Z1)	1.35
8	Peptidoglycan-binding protein (A0A0S3AGT0)	1.47	Cytochrome c-552 (P95339)	1.66	Rubrerythrin (Q2Y7X9)	1.23
9	Rubrerythrin (A0A0S3AFT3)	1.22	Bacterial outer membrane protein (Q82S16)	1.60	General diffusion Gram-negative porin (Q2Y5X1)	1.13
10	Cytochrome C (A0A0S3AHP2)	1.15	Elongation factor Tu (Q81ZS3)	1.29	Elongation factor Tu (Q2YAZ9)	0.96


*^∗^Determined by averaging the NSAF values of the triplicate ammonia replete cultures. Accession numbers are shown in brackets after protein description.*

### Significant Differences in Expression of Proteins Involved in Ammonia Oxidation

**FIGURE 1:**
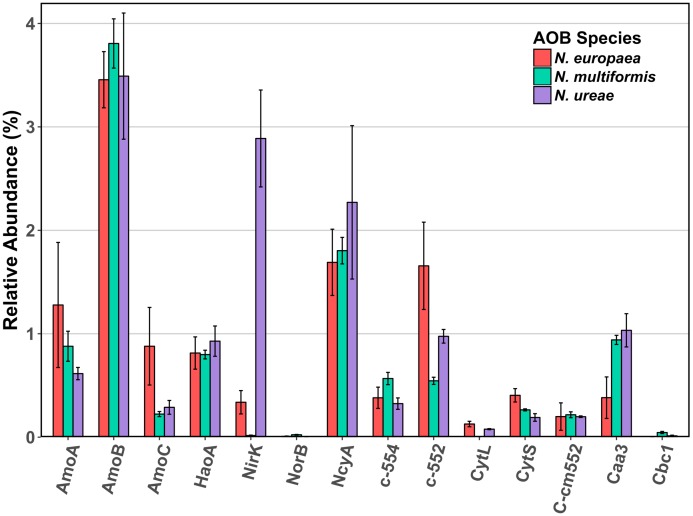
Mean relative abundance (%) of select enzymes involved in ammonia oxidation and electron transport in the proteomes of three AOB species under ammonia replete conditions. Error bars indicate SD of triplicate experiments. When multiple copies of a gene were expressed, their abundances were summed. Accession numbers for each of the enzymes for each species are listed in Supplementary Table 2. AmoA: ammonia monooxygenase subunit A, AmoB: ammonia monooxygenase subunit B, AmoC: ammonia monooxygenase subunit C, HaoA: hydroxylamine dehydrogenase, NirK: nitrite reductase, NorB: nitric oxide reductase, NcyA: nitrosocyanin, c-552: cytochrome c-552, c-554: cytochrome c-554, CytL: cytochrome P460, CytS: cytochrome c′ beta, c_m_552: cytochrome c_m_-552, caa3: cytochrome aa_3_, cbc1: cytochrome bc_1_.

The AMO was highly expressed in all three strains, which was unsurprising given the central role of this enzyme in the ammonia-oxidation pathway. Specifically, the beta subunit (AmoB) was the most highly expressed of the three subunits (*N. europaea*: 3.46%; *N. ureae*: 3.49%; *N. multiformis*: 3.81%) (**Figure 1**). The beta subunit is thought to form part of a complex with subunit A (AmoA), although its functional role is still not entirely clear (McTavish et al., 1993; Guo et al., 2013). AmoA contains the active site of the enzyme (Hyman and Wood, 1985), as further evidenced by the resolved structure of the related particulate methane monooxygenase (Lieberman and Rosenzweig, 2005), and was the next most highly expressed subunit (*N. europaea*: 1.28%, *N. multiformis*: 0.88%, and *N. ureae*: 0.61%). Subunit C (AmoC) was also highly expressed, and is thought to function as a chaperone as well as stabilizing the AMO subunits under stress-response (Klotz et al., 1997; Berube and Stahl, 2012).

The HAO was another highly expressed metabolic enzyme, but to a lesser extent than AMO in all three strains (*N. europaea*: 0.81%; *N. ureae*: 0.93%; *N. multiformis*: 0.80%). Recent evidence suggests that, contrary to previous literature, HAO catalyzes a three electron oxidation of NH_2_OH to NO, instead of a four electron oxidation to NO_2_^-^ (Caranto and Lancaster, 2017). Thus, there is likely a third enzyme involved in the ammonia oxidation process that completes the oxidation of NO to NO_2_^-^. Such an enzyme would likely be highly expressed and present in all AOB species.

**FIGURE 2:**
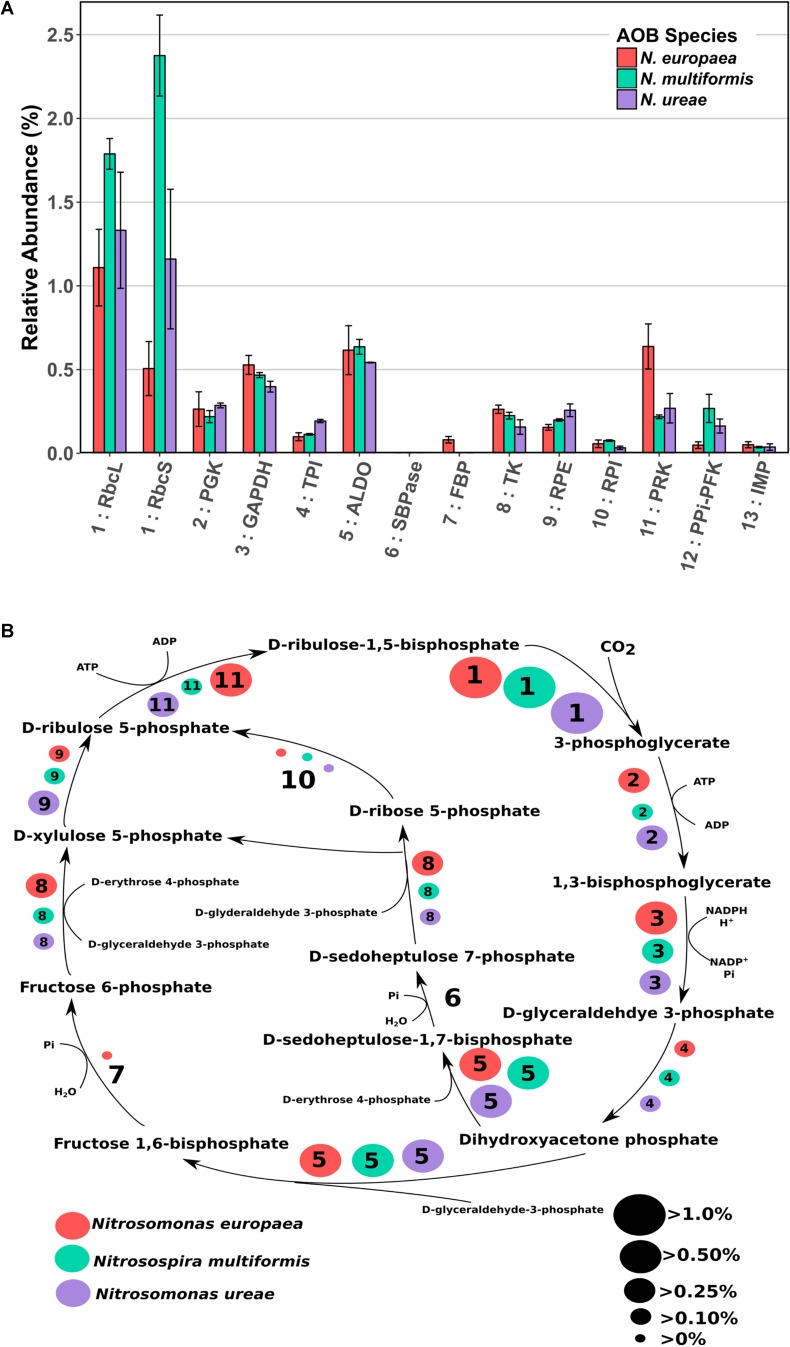
**(A)** Mean relative abundance (%) of CBB cycle enzymes in the proteomes of three AOB species under ammonia replete conditions. Error bars indicate SD of triplicate experiments. If a species had two expressed copies of a particular enzyme (see Supplementary Table 3) expression values were summed. Numbers preceding enzyme name are matched to their respective reaction in **B**. RbcL: RuBisCO large chain, RbcS: RuBisCO small chain, PGK: Phosphoglycerate kinase, GAPDH: Glyceraldehyde-3-phosphate dehydrogenase, TPI: Triosephosphate isomerase, ALDO: Fructose-bisphosphate aldolase, SBPase: Sedoheptulose bisphosphatase, FBP: Fructose-1,6-bisphosphatase, TK: Transketolase, RPE: Ribulose-phosphate-3-epimerase, RPI: Ribose-5-phosphate isomerase, PRK: Phosphoribulokinase, PPi-PFK: Pyrophosphate phosphofructokinase, IMP: Inositol monophosphatase/type IV F1P6Pase. PPi-PFK and IMP are suggestions from the literature for enzymes that could potentially complete the CBB cycle in AOB. **(B)** CBB cycle with relative abundances (%) of enzymes depicted by size of corresponding circle. Numbered circles are linked to the corresponding enzyme number depicted in **A**. Red circles indicate the abundance in *N. europaea*, teal circles indicate the abundance in *N. multiformis*, and purple circles indicate the abundance in *N. ureae*. Enzyme 6: SBPase is missing from all species, and enzyme 7: FBP is either missing, or expressed to a low amount (*N. europaea*).

From our results, another highly expressed metabolic protein apart from AMO and HAO was the red copper protein, NcyA (**Figure 2**), which was expressed to between 1.7 and 2.3% of the proteomes (**Table 1**). Previous studies of AOB in aerobic growth conditions also found NcyA in high protein concentrations and transcript levels, similar to the concentrations of other key components of the ammonia oxidizing system (Whittaker et al., 2000; Klotz and Stein, 2011). Due to its abundance, it has been previously proposed that NcyA is part of the central ammonia oxidation pathway either with an enzymatic function, or through mediating electron transfer (Arciero et al., 2002; Basumallick et al., 2005; Klotz and Stein, 2011). Specifically, it has been suggested that NcyA participates in recycling electrons from the quinone pool to AMO or functions as a relay for electrons from hydroxylamine to oxygen (Arp et al., 2007). Other specific hypotheses of roles include a functional link to nitrite reductase (Arciero et al., 2002), or a role in NO binding and reduction to N_2_O (Basumallick et al., 2005). Increased expression was previously found when *N. europaea* cultures were exposed to NO (Schmidt et al., 2004), and the protein has also been linked to the ammonia starvation response (Klotz and Stein, 2011). However, the latter role was not supported by the results of this study as there was no significant difference in expression levels of NcyA between control and starved conditions in any species (NSAF values starved cultures: replete cultures; 2.27:1.69% *N. europaea* (Q820S6), 2.49:2.27% *N. ureae* (A0A0S3AFA2), 1.99:1.80% *N. multiformis* (Q2Y8L9), *p* > 0.05, *t*-test). Given its expression at the protein level and structural potential for NO binding and enzymatic activity, it seems a likely candidate for the missing nitric oxide oxidase in AOB, although this hypothesis awaits further testing. One case against this hypothesis is that the genome of *Nitrosomonas sp. Is79* lacks the *ncyA* gene, putting into question its universality as a central metabolic enzyme in AOB (Bollmann et al., 2013). Regardless, more experiments are needed to elucidate the function of this highly expressed protein.

Another potential candidate NO oxidase is a reversely operating nitrite reductase, NirK, however, our results showed minimal expression of NirK in *N. multiformis* (0.02%) and several AOB, like *Nitrosomonas communis*, lack the *nirK* gene (Cantera and Stein, 2007; Kozlowski et al., 2016c), again calling into question its universality as a NO oxidase. Furthermore, only one study has shown the potential for NirK to operate as a NO oxidase rather than as a nitrite reductase (Wijma et al., 2004), making this enzyme an unlikely candidate as a central participant in the ammonia oxidation pathway of AOB.

Of interest, the highest NirK expression from the three species was found in *N. ureae* where the enzyme was the second most highly expressed protein (2.89%; A0A0S3AFD2). Its non-orthologous equivalents in the other two strains were expressed to a much lower level (*N. europaea*: 0.34% Q82TG8; *N. multiformis*: 0.02% Q2Y7H8), and this disparity in expression patterns is one of the most pronounced differences between the nitrogen metabolism enzymes of these three AOB (**Figure 1**). NirK is the enzyme responsible for reducing nitrite (NO_2_^-^) to NO (Wrage et al., 2001; Stein, 2011; Kozlowski et al., 2016a), and in AOB, NirK was shown to play a role in aiding in the efficient oxidation of NH_3_ to NO_2_^-^, as opposed to functioning in nitrifier denitrification (Kozlowski et al., 2014). AMO can have a higher turnover rate than HAO, and thus when excess NH_3_ is oxidized, the intermediate NH_2_OH can accumulate. NirK in AOB can aid HAO in oxidizing NH_2_OH by alleviating the electron flow bottle-neck through transferring electrons from the cytochrome pool onto NO_2_^-^. A possible explanation for the increased amounts of NirK in *N. ureae* is that the strain is generally considered oligotrophic, and although it is able to grow at the ammonia concentrations used in this study (Kozlowski et al., 2016a), *N. ureae* may not be naturally adapted to these conditions. *N. ureae* grown at NH_3_-concentrations higher than it is adapted (i.e., > 5 mM) likely causes an imbalance between the rates of NH_3_ and NH_2_OH-oxidation, which AOB adapted to higher NH_3_ concentrations are not likely to experience at the NH_3_ concentration (10 mM) used in this study. Thus, *N. ureae* could potentially be using NirK to speed the oxidation of excess NH_2_OH.

Nitric oxide reductase is a key enzyme in the AOB nitrifier denitrification pathway, converting NO to nitrous oxide (N_2_O), a potent greenhouse gas much stronger than carbon dioxide. Homologs of the nitric oxide reductases NorB and NorY are present in all AOB except for *N. ureae*, and *Nitrosomonas sp. Is79* (Bollmann et al., 2013; Kozlowski et al., 2016b). The NorB enzyme was expressed at very low levels in *N. multiformis* and *N. europaea* (**Figure 1**). The low to negligible expression of NorB in these AOB may be due to a decrease in nitrifier denitrification under the growth conditions in this study.

Cytochrome P460 (CytL) has been implicated in the oxidation of NH_2_OH and NO to NO_2_^-^ (Elmore et al., 2007; Stein, 2011), and more recently in the direct oxidation of NH_2_OH to N_2_O, producing NO_2_^-^ only as a side product when NO dissociates from the active site and reacts abiotically with oxygen (Caranto et al., 2016). In the former hypothesis, CytL was thought to be important for alleviating nitrosative stress in AOB that lack nitric oxide reductase, such as *N. ureae* (Kozlowski et al., 2016a). CytL was relatively highly expressed in *N. europaea* (H2VFU9; 0.13%), moderately expressed in *N. ureae* (A0A0S3AFC9; 0.08%), and is absent in *N. multiformis* (Norton et al., 2008). Our finding of a higher expression of CytL in *N. europaea*, which has a nitric oxide reductase, as compared to *N. ureae*, suggests that the primary role of CytL is unrelated to the absence of nitric oxide reductase under these conditions.

Other cytochromes implicated in the energy metabolism of AOB were also expressed (**Figure 1**). Cytochrome aa3 was expressed relatively highly in all three species (*N. europaea*: 0.38%, *N. multiformis*: 0.94%, and *N. ureae*: 1.03%). Cytochrome bc1 was not expressed in *N. europaea*, and was expressed at 0.05% and 0.01% in *N. multiformis*, and *N. ureae*, respectively. Cytochrome cm-552 and cytochrome c-552 were also relatively highly expressed in all three species (>0.2%). Cytochrome c-554 was very highly expressed in *N. europaea* (1.7%, Q57142), compared to *N. ureae* (0.98%, A0A0S3AN35), and *N. multiformis* (0.54%, Q2YA34).

### Proposal of Novel Enzyme Substitution for Missing Calvin-Benson-Bassham Cycle Enzymes of AOB

As autotrophs, AOB almost exclusively use the CBB cycle to acquire carbon. However, due to a few missing enzymes in AOB species, the CBB cycle in these organisms appears to deviate from the classical version (Chain et al., 2003; Norton et al., 2008; Kozlowski et al., 2016b). *N. europaea* lacks one enzyme necessary for the classical version of the CBB cycle, sedoheptulose-1,7-bisphosphatase (SBPase), whereas *N. multiformis* and *N. ureae* lack two enzymes; fructose-1,6-bisphosphatase (FBP) and SBPase (**Figure 2** and Supplementary Table 3). Potential alternative enzymes that could fulfill these missing roles in AOB have been suggested (Norton et al., 2008; Kleiner et al., 2012). For the missing SBPase, Chain et al. (2003) suggested that in *N. europaea* it could be replaced by the FBP, which is known to frequently operate as a dual function FBP/SBPase enzyme in bacteria (Yoo and Bowien, 1995; Shively et al., 1998; Miyagawa et al., 2001). For the missing FBP in *N. multiformis*, Norton et al. (2008) suggested that it could be replaced by genes that have some similarity to archaeal inositol monophosphatase/type IV FBP (IMP/FBP). They specifically identified the gene Nmul_A2147 (Q2Y731) as the most likely candidate for FBP replacement, because of similarities in domain structure and active site residues. Lastly, Kleiner et al. (2012) suggested that the missing enzymatic reactions could be performed by pyrophosphate-dependent 6-phosphofructokinase (PPi-PFK). This hypothesis is based on the fact that the PPi-PFK of *Methylococcus capsulatus*, which is highly similar to the one in AOB (75% nucleotide sequence similarity in *N. europaea* and *N. multiformis*, and 77% in *N. ureae*) was experimentally shown to catalyze reactions analogous to the FBP and SBPase reactions in a pyrophosphate-dependent manner (Reshetnikov et al., 2008). Thus PPi-PFK could enable the completion of the CBB cycle in the AOB missing two CBB cycle enzymes.

To gain a better understanding of how the CBB cycle functions in AOB we did a careful analysis of CBB cycle enzyme expression. Enzymes of the CBB cycle were highly expressed in all three species, and each enzyme accounted for between 0.03 and 2.3% of each species’ proteome, with the total expression of all CBB cycle enzymes accounting for 4.3, 6.3, and 4.6% percent of the proteomes of *N. europaea*, *N. multiformis*, and *N. ureae*, respectively. The key enzyme of the CBB cycle, RuBisCO, including both small and large subunits, was the most abundant CBB cycle enzyme (**Figure 2A**). One copy of each RuBisCO large and small subunit was expressed in *N. europaea* and *N. multiformis*, and two copies of each were expressed in *N. ureae*. Of the two copies in *N. ureae*, one pair (A0A0S3AHX5, A0A0S3AHZ4) was orthologous to the subunits from *N. multiformis*, corresponding to RuBisCO Form IC. The other RuBisCO pair (A0A0S3AFL1, A0A0S3AFG4) was orthologous to the subunits from *N. europaea* RuBisCO form IAq. The RuBisCO Form IC, which was more highly expressed by *N. ureae* in this study, is thought to have a slightly lower affinity for CO_2_ compared to RuBisCO Form IAq (Badger and Bek, 2008). The RuBisCO sequences in *N. ureae* resemble the RuBisCO copies found in the closely related *Nitrosomonas sp. Is79*, and suggests that these *Nitrosomonas* species have higher flexibility with respect to CO_2_ availability in their environments compared to other AOB species (Bollmann et al., 2013). The enzyme that was consistently the lowest expressed from the CBB cycle (<0.08%) was ribose-5-phosphate isomerase (RPI), which converts D-ribose-5 phosphate to D-ribulose-5 phosphate (**Figure 2**). In general, specific enzymes were expressed to similar levels across the species, with exceptions being phosphoribulokinase (PRK) which was expressed approximately twofold higher in *N. europaea*, and triosephosphate isomerase (TPI) which was expressed roughly twofold higher in *N. ureae*. Both sets of comparisons were significant (*p* < 0.05) with a pairwise *t*-test (Supplementary Tables 7–9).

It would be expected that the alternate enzymes performing the missing CBB cycle reactions in the three AOB species would be expressed to levels comparable to the traditional CBB cycle enzymes. The first proposed enzyme replacement was FBP, which was suggested to perform both its own function as well as that of SBPase. This functional overlap may be possible in *N. europaea* (although FBP was not expressed highly at 0.08%, in comparison with most other CBB cycle enzymes), however, this replacement enzyme is not present in *N. multiformis* or *N. ureae*. For the specific IMP/FBP (Q2Y731) proposed by Norton et al. (2008) as a SBPase and FBP substitute, expression was undetectable in *N. multiformis* in this study. Furthermore, other inositol monophosphatases present in *N. ureae* (A0A0S3AHX4, A0A0S3AIS2) and *N. multiformis* (Q2YB92), which bear some similarity to the IMP/FBP, accounted for on average, less than 0.035% of their proteomes, making them much less abundant than all other CBB cycle enzymes. *N. europaea* also has a copy of inositol monophosphatase (Q82TE3), and it was expressed at 0.05% of the proteome.

The PPi-PFK is a likely candidate for a FBP/SBPase substitute, based on the proteome expression data obtained in this study, and the fact that the PPi-PFK enzyme is able to perform the two missing reactions. PPi-PFK was expressed (0.05% in *N. europaea*, 0.16% in *N. ureae*, and 0.27% in *N. multiformis*) at levels comparable to the other CBB cycle enzymes (**Figure 2A**). In addition, *N. europaea*, which does contain FBP, showed lower expression of PPi-PFK than the other two species.

It has further been suggested that the use of PPi-PFK in the CBB cycle has the potential to save 10% of the energy in comparison with the classical version of the CBB cycle, when PPi-PFK is used in conjunction with a proton-translocating pyrophosphatase (HPPase) (Kleiner et al., 2012). The HPPase and the PPi-PFK form an operon in the genomes of all three analyzed AOB species, which indicates a tight metabolic coupling between the two enzymes. Hydrolysis of PPi, which is produced by PPi-PFK when cleaving the phosphate of sedoheptulose-1,7-bisphosphate and fructose-1,6-bisphosphate, by the HPPase would lead to energy conservation in form of a proton motive force. All three species expressed HPPase [0.17% in *N. europaea* (Q82TF3), 0.13% in *N. ureae* (A0A0S3AI31), and 0.10% in *N. multiformis* (Q2YB25)], indicating that a PPi-dependent energy economy plays a role in AOB. In summary, our proteomic data supports the hypothesis that PPi-PFK substitutes for FBP and SBPase in the CBB cycle of AOB and that the CBB cycle of AOB may be more energy efficient due to PPi-dependent energy conservation.

### Major Differences in Expression of Orthologous Genes Across the AOB

Using the program Orthovenn (Wang et al., 2015), we identified orthologous protein encoding genes shared between either two or all three AOB species, as well as genes that are unique to individual species. By comparing the expression of orthologous proteins, we could directly determine differences in the allocation of cellular resources toward cellular functions. Of all the protein encoding genes, 1253 were identified as having orthologs in all three species (1207 of which only occurred in one copy per genome), 648 were considered orthologs common to two species, and 162 were found in multiple copies but only in one species. Of these unique genes, 41 were found in *N. europaea*, 57 in *N. ureae*, and 64 in *N. multiformis* (Supplementary Figure 2). The remaining genes were found in only one copy in one species and resulted in 524 singletons in *N. europaea*, 873 in *N. ureae*, and 825 in *N. multiformis*.

To identify orthologous proteins that differed in expression between species, we applied pairwise *t*-tests (*p* < 0.05) corrected for multiple testing with permutation based FDR to all culture replicates, and detected hundreds of statistically significant differences. A comprehensive list of all orthologous proteins and the corresponding statistical information is presented in Supplementary Tables 7–9 and Supplementary Table 4 provides a description of each of the tables’ contents. A total of 798 proteins out of the 1207 single gene orthologs were detectable to some degree in one of the three species at the protein level. Out of these 798 proteins, 585 proteins were statistically differentially expressed between *N. europaea* and *N. multiformis* (73% of three species orthologs), 626 proteins were statistically differentially expressed between *N. multiformis* and *N. ureae* (78% of three species orthologs), and 508 proteins were statistically differentially expressed between *N. ureae* and *N. europaea* (64% of three species orthologs). *N. europaea* and *N. multiformis* shared 90 two-species orthologs, and out of these 80 were differentially expressed. *N. multiformis* and *N. ureae* shared 94 two-species orthologs, and out of these 87 were expressed differentially. *N. europaea* and *N. ureae* shared 58 two-species orthologs, and out of these 46 were expressed differentially.

**FIGURE 3:**
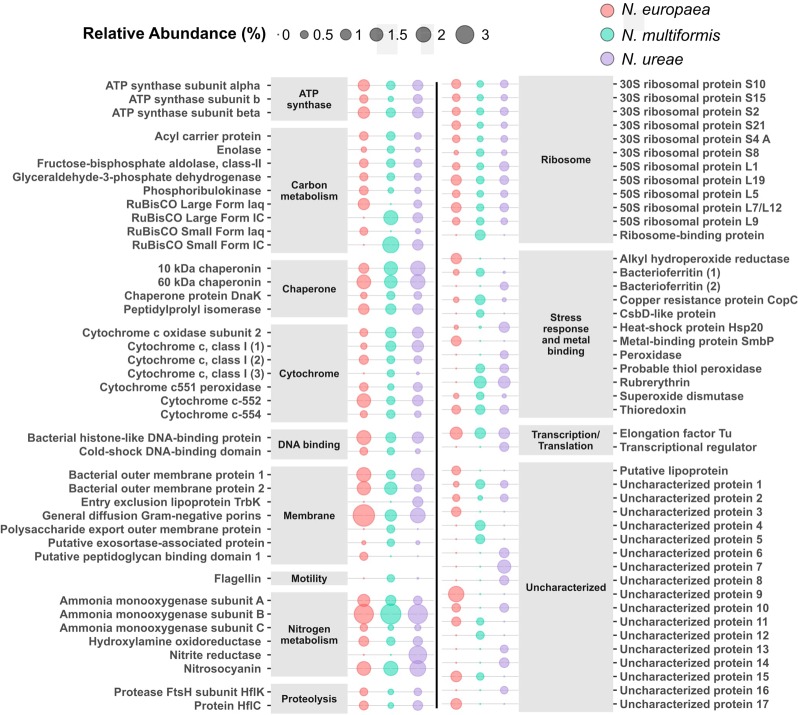
Top 50 most abundant proteins in each species with orthologs from the other species. Supplementary Table 5 contains accession numbers for each protein. Size relates to relative abundance (%) in the proteome of each species.

The betaproteobacteria AOB as a tight phylogenetic group offer a compelling system to examine how functionally similar microbes make use of differential gene expression, enabling competitiveness within a range of physicochemical factors including pH, temperature, substrate concentration, oxygen tension, trace metals, and salinity among others (Bernhard et al., 2005; Fierer et al., 2009; Martens-Habbena et al., 2009; Wells et al., 2009). Prior studies comparing genome content, regulation of genes and proteins, and relative gene abundance at ecosystem levels suggest that this genomic flexibility enables each species to occupy a preferred niche (Norton, 2011). Hence, it is not surprising that many orthologous genes showed differential expression at the protein level in this study as each of the three compared species is found occupying different habitats, and thus, were expected to show a wide range of responses to identical growth conditions (Prosser et al., 2014). The top 50 most abundant proteins from each species and the abundance of orthologous proteins in the other species (if present) are shown in **Figure 3** and listed in Supplementary Table 5. Due to the large number of differences in orthologous protein expression we only highlight a few interesting examples from cellular subsystems below.

#### Motility

The three AOB species investigated have the capacity for motility with orthologous proteins encoding flagellin and flagellin regulation present in each genome. Under the present growth conditions, however, the flagellin protein was not expressed at all in *N. europaea*, and only slightly in *N. ureae* (0.006%, A0A0S3AJU9). In contrast, *N. multiformis* expressed its flagellin protein (Q2Y9D1) to a significantly higher level than the other two strains, and it accounted for 0.43% of its proteome. Additionally, the sigma factor controlling for the expression of flagella-related genes (FliA) was only expressed in *N. multiformis*. Thus, *N. multiformis* allocates significant resources to motility compared to the other two species.

#### Cell Division

The MraZ transcription regulator, involved in inhibition of cell division among other functions (Eraso et al., 2014), was more highly expressed in *N. ureae* (0.07%, A0A0S3AHI1) than the other two species (*N. europaea*: 0.005% Q82VT2, *N. multiformis*: 0.02% Q2Y629). *N. ureae* had both higher expression of the MraZ transcriptional regulator and slower growth rates than the other two species agreeing with its functional description (Supplementary Figure 1). Furthermore, there was a statistically significant relationship between generation time and MraZ expression across these AOB species (Spearman’s correlation = 0.85, *p* < 0.005, *n* = 9). In contrast to MraZ expression, the ATP-dependent zinc metalloprotease FtsH, was most highly expressed in *N. europaea* (0.13%, Q82VZ3), followed by *N. multiformis* (0.04%, Q2Y826), and at the lowest level in *N. ureae* (0.01%, A0A0S3AFK9). FtsH regulates the expression of genes required for the synthesis of cell wall lipopolysaccharide components, an important process in actively dividing cells. Correlation of FtsH expression with the generation time of the AOB cultures was highly significant (Spearman’s correlation = -0.85, *p* < 0.005, *n* = 9). Additionally, there was a negative correlation between the expression levels of MraZ and FtsH across AOB replicates (Spearman’s correlation = -0.87, *n* = 9, *p* = 0.003, Supplementary Figure 3). FtsZ, the ubiquitous key protein involved in cell division, was significantly more highly expressed in *N. europaea* (Q820N2, 0.12%) and *N. multiformis* (Q2Y644, 0.14%) than in *N. ureae* (A0A0S3AHF2, 0.03%). The relationship between FtsZ and generation time across the species, however, was not significant (*p* > 0.05). Generation time was highly correlated with the expression of 40 additional proteins (see Supplementary Table 6 for more details). Of these proteins, the majority that were correlated with faster growing cells were related to protein synthesis or cell division (Supplementary Table 6).

#### Stress Response

Several proteins involved in AOB stress response, including nitrosative and oxidative stresses, showed strong differences in expression between the three species. Nitrosative and oxidative stress from exposure to reactive nitrogen species (RNS) and reactive oxygen species (ROS), respectively, are common in environmental bacteria like AOB. The origin of these RNS/ROS can be from interactions with other organisms, by-products of aerobic cellular metabolism, or they can be photochemically produced from organic solutes in soil and other environments (Wood and Sørensen, 2001). Rubrerythrin belongs to the ferritin-like superfamily and is involved in oxidative stress relief mainly by acting as a peroxide scavenger (Cardenas et al., 2016). Originally evolving to protect anaerobic species in increasingly aerobic conditions, this protein has since evolved to be used by aerobic organisms. Among the AOB species included in this study, *N. ureae* (A0A0S3AFT3) and *N. multiformis* (Q2Y7X9) had orthologous copies of rubrerythrin that were expressed at high levels (1.22 and 1.23% of proteomes, respectively). Interestingly, although highly expressed in the other two species, *N. europaea* does not contain an ortholog for this gene. Without a rubrerythrin copy in *N. europaea*, alternate enzymes are likely responsible for this particular oxidative stress function. One candidate is alkyl hydroxide reductase (Q820H3), a highly expressed enzyme in *N. europaea* (0.91%), which also scavenges peroxide radicals (Seaver and Imlay, 2001). The gene has one ortholog in *N. multiformis*, which was not expressed, but no orthologs in *N. ureae*.

Superoxide dismutase catalyzes the partitioning of superoxide radicals, which can form during oxygen metabolism, into either molecular oxygen or hydrogen peroxide. The enzyme was expressed to high levels in all three species, but was significantly more highly expressed in *N. multiformis* (0.39%, Q2YBR2), than *N. europaea* (0.19%, Q82W28), and *N. ureae* (0.10%, A0A0S3ALT7). Catalase, the enzyme that converts hydrogen peroxide (the potential product of superoxide dismutase) to water and oxygen was expressed to low levels (< 0.05%) in *N. europaea* (Q82TK1) and *N. ureae* (A0A0S3AIK6), while the ortholog in *N. multiformis* (Q2Y8V3) was not detected.

Heat shock protein 20 (Hsp20) was much more highly expressed in *N. ureae* (0.82%, A0A0S3AHM5) than *N. multiformis* (0.02%, Q2Y862), and *N. europaea* (0.06%, Q82VT2). Heat shock proteins are normally expressed when an organism is exposed to environmental stress, and in these conditions, they act as protein chaperones, preventing misfolding of other proteins. In the present study, the three species were exposed to the exact same growth conditions, suggesting that the high levels in *N. ureae* are either due to constitutive expression of the gene, or suboptimal growth conditions (e.g., high NH_3_ concentrations) for the strain.

The copper resistance protein CopC is important for protecting against copper toxicity, as well as for copper utilization. The *copCD* genes are located within the *amoCAB* gene cluster, indicating functional relatedness to the AMO enzyme. CopC was more highly expressed in *N. multiformis* (0.70%, Q2Y5C3) than *N. europaea* (0.18%, Q82T65) or *N. ureae* (0.093%, A0A0S3AIQ1). Copper is in the active site of AMO and NirK and as CopC is important in copper binding and uptake, it is also essential for the functioning of these two enzymes. The other subunit to the enzyme CopD was expressed to much lower levels in all species (*N. europaea*: 0.02% Q820J3, *N. multiformis*: 0.03% Q2Y5C4, and not expressed in *N. ureae*).

#### Urea Utilization

Under certain conditions (i.e., low pH environments), urea can be used as both a source of nitrogen and carbon in some AOB (Marsh et al., 2005). *N. ureae*, as its name suggests, is one of the AOB species capable of using urea (Koops et al., 1991). The three subunits of the enzyme urease, which converts urea and water to carbon dioxide and ammonia, were moderately expressed in *N. ureae* (0.06–0.11%). Urease was barely expressed in *N. multiformis* (less than 0.02%) and *N. europaea* lacks a homologous gene (Chain et al., 2003). Urea carboxylase, which catalyzes the reaction of urea, ATP, and bicarbonate to urea-1-carboxylate (allophanate), was represented by three copies (A0A0S3AHN5, A0A0S3AI24, and A0A0S3AHN2) in the *N. ureae* proteome, but none of the three were expressed to greater than 0.04%. Orthologs of urea carboxylase were present in the other two genomes but were undetectable at the protein level. Low expression of urea related proteins is to be expected as no urea was added to the media, however, the observed consistent expression of urease in *N. ureae* suggests it could be constitutively expressed in this species.

#### Uncharacterized Proteins Without Orthologs

Many uncharacterized proteins without orthologs in the other two species (singletons) were highly expressed at the protein level. For instance, Q2Y9X6, Q2Y9X8 and Q2Y844 (DUF2795 domain) were expressed to 0.81, 0.69, and 0.37% of the proteome, respectively, from *N. multiformis*. From *N. ureae*, uncharacterized singleton proteins A0A0S3AIB4, A0A0S3AFI8, and A0A0S3AKF1 were expressed to 1.5, 0.78, and 0.71%, respectively. *N. europaea* also had uncharacterized singleton proteins Q82VY9 (PepSY domain), Q82V70 (DUF533 domain), and Q82TW7 (LTXXQ motif) highly expressed to 0.75, 0.38, and 0.23% of the proteome. High expression of uncharacterized proteins without orthologs in these AOB suggests that more work is needed in elucidating the function of hypothetical proteins with potentially important roles central to AOB metabolism.

### Response to Ammonia Starved Conditions

Overall, surprisingly few proteins exhibited a statistically significant change in expression in response to 24 h of ammonia starvation. Assays confirmed that the control cultures contained ammonia and that the starved cultures did not. Furthermore, after 24 h of incubation, the production of nitrite had started in the control cultures, suggesting active ammonia oxidation, but no nitrite was measured in the starved cultures (Supplementary Table 1). The control cultures had accumulated 0.25–1 mM of nitrite, and these values are comparable to the daily nitrite production observed in pre-cultures. We also performed correlation analysis between substrate concentrations after 24 h (nitrification activity) and relative protein abundances and found no significant relationships. The lack of response to a full day of ammonia starvation in the AOB species tested shows that these AOB regulate their proteomes similarly despite ecological differences. Important to point out, however, is that in the present study we aimed to identify changes in the relative abundance of proteins, but we cannot rule out the possibility of alternate regulation of protein function in the form of post-translational modifications, or substrate level activation or inhibition.

*Nitrosospira multiformis* was the only strain in which proteins showed statistically significant differences between control and starved cultures. These differences were minor, however, as from the 1064 proteins expressed by this strain only three were identified as statistically significant using *t*-tests (*p* < 0.05) after correcting for multiple tests using permutation based FDR. The differentially expressed proteins were heat shock protein Hsp20 (Q2Y862, 0.1% average in starved compared to 0.02% average in control), RND efflux system outer membrane lipoprotein NodT (Q2Y897, 0.006% average in starved compared to 0.02% average in control), and RuBisCO large chain (Q2YB78, 0.96% average in starved compared to 1.8% average in control).

Steady expression levels of AMO throughout the experiment were a strong indication of a slow functional response to ammonia starvation by these organisms. In support of this, it has been suggested based on similar observations in prior studies that *N. europaea* cells maintain a basal level of AMO enzyme activity that is largely insensitive to changes in ammonia concentration (Stein et al., 1997; Geets et al., 2006). This basal level of AMO expression in AOB could present an immediate advantage to the cells when ammonia is available again and could be a strategy indicative of cells adapted to substrate fluctuations.

A proteomics study by Pellitteri-Hahn et al. (2011) identified only 27 proteins that were differentially expressed after a 2-week period of ammonia starvation in *N. europaea*, significantly longer than the present study. The proteins with greater abundance in growing cells from that study were geared toward biosynthesis, while energy starved cells had more proteins related to survival functions. In contrast, a study that looked at the starvation response of *N. europaea*, through transcriptomics and with respect to both ammonia and carbonate, saw 90% of transcripts present at twofold greater levels in growing cells compared to cells that had been deprived of ammonia for only 16 h (Wei et al., 2006). This suggests that at least in some AOB there is a relatively quick regulatory response to ammonia depletion on the level of transcription likely leading to a decrease in production of new protein. Consequently, the energy that was invested in initial protein synthesis is conserved in AOB. Keeping protein machinery intact would allow these species the opportunity to quickly respond to increases in nutrients. This hypothesis is also supported by previous studies that observed extremely quick renewal of NH_3_-oxidation activity of AOB following starvations that varied in length from weeks to many months (Wilhelm et al., 1998; Tappe et al., 1999; Bollmann et al., 2005). Furthermore, a recent study monitoring the response of *N. europaea* to daily anoxic-oxic cycles found that short-term responses (a few hours) to stress were regulated at the level of mRNA, whereas long-term responses (after 12 days) to the same stress were regulated at the level of proteins (Yu et al., 2018). Thus, our finding of a minimal response at the protein level to ammonia starvation after 24 h is in agreement with the recent study by Yu et al. (2018).

**FIGURE 4:**
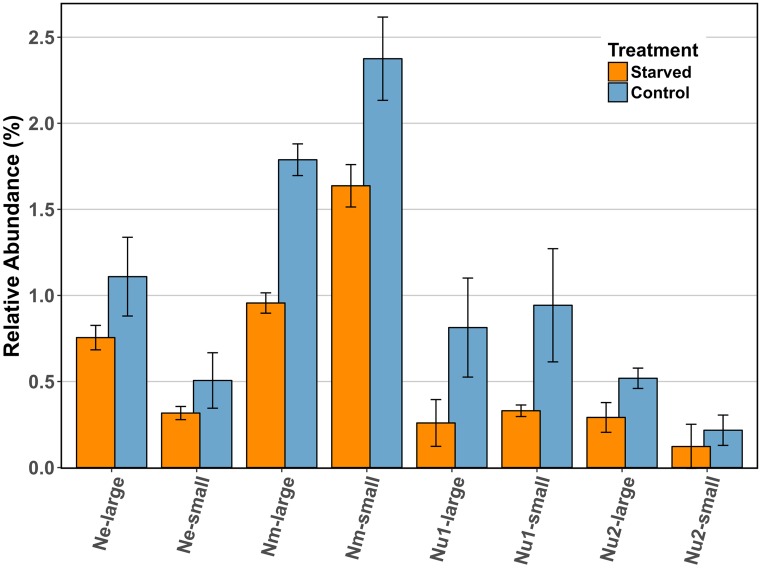
Comparison of the relative abundance (%) of RuBisCO large and small subunits across species in control and starved conditions. Ne: *N. europaea*, Nm: *N. multiformis*, and Nu: *N. ureae*. Nu1 refers to the copy of *N. ureae* RuBisCO subunits that are orthologous to *N. multiformis* RuBisCO subunits (A0A0S3AHX5, A0A0S3AHZ4), while Nu2 refers to the other copy, orthologous to *N. europaea* RuBisCO subunits (A0A0S3AFL1, A0A0S3AFG4). Only *N. multiformis* RuBisCO large subunit (Nm-large) was identified as statistically significant with a *t*-test corrected for FDR.

Despite the absence of a coordinated starvation response, the RuBisCO enzyme consistently decreased in abundance in starved cells as compared to control cells in all three species (**Figure 4**), and this decrease was statistically significant for the large subunit of *N. multiformis*. In *N. multiformis* the ratio of starved to control for the large subunit was 0.53, in *N. europaea* it was 0.68, and in *N. ureae* it was 0.32 and 0.56 for the two expressed copies. The small subunit of RuBisCO also decreased between starved and control conditions. In *N. multiformis* the ratio was 0.69, in *N. europaea* the ratio was 0.63, and in *N. ureae* the ratio was 0.35 and 0.56. In a previous starvation study using transcript abundance, RuBisCO transcripts were found to decrease significantly in ammonia starved conditions, and they decreased to a greater extent than *amoCAB* and *haoAB* transcript levels (Wei et al., 2004). Additionally, in a study observing the transcriptome of *Nitrosococcus oceani*, an AOB from the class of Gammaproteobacteria, transcripts involved in carbon fixation (i.e., RuBisCO) strongly decreased in response to a change in energy status due to ammonia starvation (Stein et al., 2013). RuBisCO catalyzes the rate limiting step of the CBB cycle, which often behaves as the ultimate electron sink in chemolithoautotrophic bacteria (Bar-Even et al., 2012). Thus, the reduction of RuBisCO in the proteome could act to immediately reduce the requirement for electrons and energy from ammonia oxidation in times of scarcity, without having to break down other CBB cycle enzymes. In this manner, the AOB are limiting anabolic metabolism by specifically targeting the enzyme that best controls these processes. Tappe et al. (1999) suggested that in non-spore forming bacteria, maintenance energy demand during periods of starvation should be as low as possible, but still sufficient to ensure a fast response when nutrients become available again (Tappe et al., 1999). Maintenance energy is defined as the energy consumed during the activities that allow the cell to survive without biomass production. As RuBisCO and the CBB cycle are responsible for the build-up of biomass in AOB, the reduction of RuBisCO in the proteome is in line with this hypothesis.

## Conclusion

In summary, a 24-h period of ammonia starvation did not trigger a robust response in the proteomes of the three AOB species analyzed in this study. This suggests that even with the ecological differences between the species, they respond to fluctuations in substrate availability in this time-period in a similar manner at the protein level. We would like to suggest, however, that longer starvation times and the corresponding shift in the AOB proteome should be explored in the future as the AOB response to these changes may be slow (Yu et al., 2018). Although all AOB perform the same core function of ammonia oxidation to nitrite, they inhabit a variety of environments, from the oligotrophic ocean to eutrophic wastewater treatment plants, sometimes with multiple AOB species coexisting in the same environment (Gao et al., 2014). There were major differences in the presence and expression of a large number of orthologous genes between the three species, likely representing adaptations to these different environments and partitioning of the AOB species into their ecological niches (Norton, 2011). From the present study, various other insights were gained regarding how AOB allocate resources at the protein level. For instance, enzymes of the central energy-generating NH_3_-oxidation pathway, such as AMO, are highly expressed in all three species, whereas other enzymes in this pathway, like NirK, are expressed at significantly different levels, suggesting differing responses among the AOB to similar conditions and the potential for alternate flux of intermediates in the pathway.

## Data Deposition

The mass spectrometry proteomics data have been deposited to the ProteomeXchange Consortium via the PRIDE (Vizcaíno et al., 2016) partner repository with the dataset identifier PXD008954.

## Author Contributions

JZ conceived study, planned, and performed the starvation experiments, prepared the samples for mass spectrometry, analyzed the mass spectrometric data, and wrote the paper with input from all co-authors. JK revised the manuscript, provided the expertise in the field of AOB, and aided in the analysis of mass spectrometric data. LS revised the manuscript, provided the expertise in the field of AOB, and aided in the analysis of mass spectrometric data. MS conceived the study, revised the manuscript, and aided in the analysis of mass spectrometric data. MK conceived the study, helped plan the experiments, helped in the generation of mass spectrometric data, aided in the analysis of mass spectrometric data, and revised the manuscript.

## Conflict of Interest Statement

The authors declare that the research was conducted in the absence of any commercial or financial relationships that could be construed as a potential conflict of interest.

## Footnotes

1 http://www.thegpm.org/crap/

## References

[B1] Aitchison J. (1982). The statistical analysis of compositional data. 44 139–177. 10.2307/2345821

[B2] Arciero D. M. Pierce B. S. Hendrich M. P. Hooper A. B. (2002). Nitrosocyanin, a red cupredoxin-like protein from *Nitrosomonas europaea*. 41 1703–1709. 10.1021/bi015908w 11827513

[B3] Arp D. J. Chain P. S. G. Klotz M. G. (2007). The impact of genome analyses on our understanding of ammonia-oxidizing bacteria. 61 503–528. 10.1146/annurev.micro.61.080706.093449 17506671

[B4] Badger M. R. Bek E. J. (2008). Multiple Rubisco forms in proteobacteria: their functional significance in relation to CO2 acquisition by the CBB cycle. 59 1525–1541. 10.1093/jxb/erm297 18245799

[B5] Bar-Even A. Noor E. Milo R. (2012). A survey of carbon fixation pathways through a quantitative lens. 63 2325–2342. 10.1093/jxb/err417 22200662

[B6] Basumallick L. Sarangi R. DeBeer George S. Elmore B. Hooper A. B. Hedman B. (2005). Spectroscopic and density functional studies of the red copper site in nitrosocyanin: role of the protein in determining active site geometric and electronic structure. 127 3531–3544. 10.1021/ja044412+ 15755175

[B7] Bernhard A. E. Donn T. Giblin A. E. Stahl D. A. (2005). Loss of diversity of ammonia-oxidizing bacteria correlates with increasing salinity in an estuary system. 7 1289–1297. 10.1111/j.1462-2920.2005.00808.x 16104852

[B8] Berube P. M. Stahl D. A. (2012). The divergent AmoC3 subunit of ammonia monooxygenase functions as part of a stress response system in *Nitrosomonas europaea*. 194 3448–3456. 10.1128/JB.00133-12 22544266 PMC3434715

[B9] Bollmann A. Laanbroek H. (2001). Continuous culture enrichments of ammonia-oxidizing bacteria at low ammonium concentrations. 37 211–221. 10.1016/S0168-6496(01)00163-5

[B10] Bollmann A. Laanbroek J. (2002). Growth at low ammonium concentrations and starvation response as potential factors involved in niche differentiation among ammonia-oxidizing bacteria. 68 4751–4757. 10.1128/AEM.68.10.4751 12324316 PMC126422

[B11] Bollmann A. Schmidt I. Saunders A. M. Nicolaisen M. H. (2005). Influence of starvation on potential ammonia-oxidizing activity and amoA mRNA levels in *Nitrosospira briensis*. 71 1276–1282. 10.1128/AEM.71.3.1276-1282.2005 15746329 PMC1065156

[B12] Bollmann A. Sedlacek C. J. Norton J. Laanbroek H. J. Suwa Y. Stein L. Y. (2013). Complete genome sequence of *Nitrosomonas sp. Is79*, an ammonia oxidizing bacterium adapted to low ammonium concentrations. 7 469–482. 10.4056/sigs.3517166 24019993 PMC3764937

[B13] Cantera J. J. L. Stein L. Y. (2007). Molecular diversity of nitrite reductase genes (nirK) in nitrifying bacteria. 9 765–776. 10.1111/j.1462-2920.2006.01198.x 17298375

[B14] Caranto J. D. Lancaster K. M. (2017). Nitric oxide is an obligate bacterial nitrification intermediate produced by hydroxylamine oxidoreductase. 114 8217–8222. 10.1073/pnas.1704504114 28716929 PMC5547625

[B15] Caranto J. D. Vilbert A. C. Lancaster K. M. (2016). *Nitrosomonas europaea* cytochrome P460 is a direct link between nitrification and nitrous oxide emission. 113 14704–14709. 10.1073/pnas.1611051113 27856762 PMC5187719

[B16] Cardenas J. P. Quatrini R. Holmes D. S. (2016). Aerobic lineage of the oxidative stress response protein rubrerythrin emerged in an ancient microaerobic, (hyper)thermophilic environment. 7:1822. 10.3389/fmicb.2016.01822 27917155 PMC5114695

[B17] Chain P. Lamerdin J. Larimer F. Regala W. Lao V. Land M. (2003). Complete genome sequence of the ammonia-oxidizing bacterium and obligate chemolithoautotroph *Nitrosomonas europaea*. 185 2759–2773. 10.1128/JB.185.9.2759 12700255 PMC154410

[B18] Daims H. Lebedeva E. V. Pjevac P. Han P. Herbold C. Albertsen M. (2015). Complete nitrification by *Nitrospira bacteria*. 528 504–509. 10.1038/nature16461 26610024 PMC5152751

[B19] Elmore B. O. Bergmann D. J. Klotz M. G. Hooper A. B. (2007). Cytochromes P460 and *c*’-beta; a new family of high-spin cytochromes *c*. 581 911–916. 10.1016/j.febslet.2007.01.068 17292891

[B20] Eraso J. M. Markillie L. M. Mitchell H. D. Taylor R. C. Orr G. Margolin W. (2014). The highly conserved MraZ protein is a transcriptional regulator in *Escherichia coli*. 196 2053–2066. 10.1128/JB.01370-13 24659771 PMC4010979

[B21] Fernandes A. D. Reid J. N. Macklaim J. M. McMurrough T. A. Edgell D. R. Gloor G. B. (2014). Unifying the analysis of high-throughput sequencing datasets: characterizing RNA-seq, 16S rRNA gene sequencing and selective growth experiments by compositional data analysis. 2:15. 10.1186/2049-2618-2-15 24910773 PMC4030730

[B22] Fierer N. Carney K. M. Horner-Devine M. C. Megonigal J. P. (2009). The biogeography of ammonia-oxidizing bacterial communities in soil. 58 435–445. 10.1007/s00248-009-9517-9 19352770

[B23] Gao J. Luo X. Wu G. Li T. Peng Y. (2014). Abundance and diversity based on amoA genes of ammonia-oxidizing archaea and bacteria in ten wastewater treatment systems. 98 3339–3354. 10.1007/s00253-013-5428-2 24318009

[B24] Geets J. Boon N. Verstraete W. (2006). Strategies of aerobic ammonia-oxidizing bacteria for coping with nutrient and oxygen fluctuations. 58 1–13. 10.1111/j.1574-6941.2006.00170.x 16958903

[B25] Griess-Romijn van Eck E. (1966). Rijswijk: Nederlands Normalisatie Instituut.

[B26] Gruber N. Galloway J. N. (2008). An Earth-system perspective of the global nitrogen cycle. 451 293–296. 10.1038/nature06592 18202647

[B27] Guo J. Peng Y. Wang S. Ma B. Ge S. Wang Z. (2013). Pathways and organisms involved in ammonia oxidation and nitrous oxide emission. 43 2213–2296. 10.1080/10643389.2012.672072

[B28] Hommes N. G. Sayavedra-Soto L. A. Arp D. J. (2003). Chemolithoorganotrophic growth of *Nitrosomonas europaea* on fructose. 185 6809–6814. 10.1128/JB.185.23.6809-6814.2003 14617645 PMC262722

[B29] Hyman M. R. Wood P. M. (1985). Suicidal inactivation and labelling of ammonia mono-oxygenase by acetylene. 227 719–725. 10.1042/BJ2270719 4004794 PMC1144898

[B30] Jiang D. Khunjar W. O. Wett B. Murthy S. N. Chandran K. (2015). Characterizing the metabolic trade-off in *Nitrosomonas europaea* in response to changes in inorganic carbon supply. 49 2523–2531. 10.1021/es5043222 25546702

[B31] Kerou M. Offre P. Valledor L. Abby S. S. Melcher M. Nagler M. (2016). Proteomics and comparative genomics of *Nitrososphaera viennensis* reveal the core genome and adaptations of archaeal ammonia oxidizers. 113 E7937–E7946. 10.1073/pnas.1601212113 27864514 PMC5150414

[B32] Kleiner M. Thorson E. Sharp C. E. Dong X. Liu D. Li C. (2017). Assessing species biomass contributions in microbial communities via metaproteomics. 8:1558. 10.1038/s41467-017-01544-x 29146960 PMC5691128

[B33] Kleiner M. Wentrup C. Lott C. Teeling H. Wetzel S. Young J. (2012). PNAS Plus: metaproteomics of a gutless marine worm and its symbiotic microbial community reveal unusual pathways for carbon and energy use. 109 E1173–E1182. 10.1073/pnas.1121198109 22517752 PMC3358896

[B34] Klotz M. G. Alzerreca J. Norton J. M. (1997). A gene encoding a membrane protein exists upstream of the amoA/amoB genes in ammonia oxidizing bacteria: a third member of the amo operon? 150 65–73. 10.1111/j.1574-6968.1997.tb10351.x 9163908

[B35] Klotz M. G. Stein L. Y. (2011). “Genomics of ammonia-oxidizing bacteria and insights into their evolution,” in , eds Ward B. B. Arp D. J. Klotz M. G. (Washington, DC: ASM Press), 57–94.

[B36] Koops H. P. Boettcher B. Moller U. C. Pommerening-Roeser A. Stehr G. (1991). Classification of eight new species of ammonia-oxidizing bacteria?. 137 1689–1699. 10.1099/00221287-137-7-1689

[B37] Kowalchuk G. A. Stephen J. R. (2001). Ammonia-oxidizing bacteria: a model for molecular microbial ecology. 55 485–529. 10.1146/annurev.micro.55.1.485 11544365

[B38] Kozlowski J. A. Kits K. D. Stein L. Y. (2016a). Comparison of nitrogen oxide metabolism among diverse ammonia-oxidizing bacteria. 7:1090 10.3389/fmicb.2016.01090 PMC4940428 27462312

[B39] Kozlowski J. A. Kits K. D. Stein L. Y. (2016b). Complete genome sequence of *Nitrosomonas ureae* strain Nm10, an oligotrophic group 6a nitrosomonad. 4 4–5. 10.1128/genomeA.00094-16.Copyright 26966201 PMC4786657

[B40] Kozlowski J. A. Kits K. D. Stein L. Y. (2016c). Genome sequence of *Nitrosomonas communis* strain Nm2, a mesophilic ammonia-oxidizing bacterium isolated from Mediterranean soil. 4:e01541–15. 10.1128/genomeA.01541-15 26769932 PMC4714114

[B41] Kozlowski J. A. Price J. Stein L. Y. (2014). Revision of N_2_O-producing pathways in the ammonia-oxidizing bacterium *Nitrosomonas europaea* ATCC 19718. 80 4930–4935. 10.1128/AEM.01061-14 24907318 PMC4135743

[B42] Krummel A. Harms H. (1982). Effect of organic matter on growth and cell yield of ammonia-oxidizing bacteria. 133 50–54. 10.1007/BF00943769

[B43] Kumar N. Bansal A. Sarma G. S. Rawal R. K. (2014). Chemometrics tools used in analytical chemistry: an overview. 123 186–199. 10.1016/j.talanta.2014.02.003 24725882

[B44] Lieberman R. L. Rosenzweig A. C. (2005). Crystal structure of a membrane-bound metalloenzyme that catalyses the biological oxidation of methane. 434 177–182. 10.1038/nature03311 15674245

[B45] Marsh K. L. Sims G. K. Mulvaney R. L. (2005). Availability of urea to autotrophic ammonia-oxidizing bacteria as related to the fate of 14C- and 15N-labeled urea added to soil. 42 137–145. 10.1007/s00374-005-0004-2

[B46] Martens-Habbena W. Berube P. M. Urakawa H. de la Torre J. R. Stahl D. A. Torre J. (2009). Ammonia oxidation kinetics determine niche separation of nitrifying Archaea and Bacteria. 461 976–979. 10.1038/nature08465 19794413

[B47] McTavish H. Fuchs J. A. Hooper A. B. (1993). Sequence of the gene coding for ammonia monooxygenase in *Nitrosomonas europaea*. 175 2436–2444. 10.1128/jb.175.8.2436-2444.1993 PMC204533 8468301

[B48] Mellbye B. L. Giguere A. Chaplen F. Bottomley P. J. Sayavedra-Soto L. A. (2016). Steady state growth under inorganic carbon limitation increases energy consumption for maintenance and enhances nitrous oxide production in *Nitrosomonas europaea*. 82:AEM.00294–16. 10.1128/AEM.00294-16 27016565 PMC4959225

[B49] Miyagawa Y. Tamoi M. Shigeoka S. (2001). Overexpression of a cyanobacterial fructose-1,6-/sedoheptulose-1,7-bisphosphatase in tobacco enhances photosynthesis and growth. 19 965–969. 10.1038/nbt1001-965 11581664

[B50] Nicol G. W. Leininger S. Schleper C. Prosser J. I. (2008). The influence of soil pH on the diversity, abundance and transcriptional activity of ammonia oxidizing archaea and bacteria. 10 2966–2978. 10.1111/j.1462-2920.2008.01701.x 18707610

[B51] Norton J. M. (2011). “Diversity and environmental distribution of ammonia-oxidizing bacteria,” in , eds Ward B. B. Klotz M. G. Arp D. J. (Washington, DC: ASM Press), 39–55. 10.1128/9781555817145.ch3

[B52] Norton J. M. Klotz M. G. Stein L. Y. Arp D. J. Bottomley P. J. Chain P. S. G. (2008). Complete genome sequence of *Nitrosospira multiformis*, an ammonia-oxidizing bacterium from the soil environment. 74 3559–3572. 10.1128/AEM.02722-07 18390676 PMC2423025

[B53] Pellitteri-Hahn M. C. Halligan B. D. Scalf M. Smith L. Hickey W. J. (2011). Quantitative proteomic analysis of the chemolithoautotrophic bacterium *Nitrosomonas europaea*: comparison of growing- and energy-starved cells. 74 411–419. 10.1016/j.jprot.2010.12.003 21172464

[B54] Pérez J. Buchanan A. Mellbye B. Ferrell R. Chang J. H. Chaplen F. (2014). Interactions of *Nitrosomonas europaea* and *Nitrobacter winogradskyi* grown in co-culture. 197 79–89. 10.1007/s00203-014-1056-1 25362506

[B55] Petersen J. M. Kemper A. Gruber-Vodicka H. Cardini U. van der Geest M. Kleiner M. (2016). Chemosynthetic symbionts of marine invertebrate animals are capable of nitrogen fixation. 2:16195. 10.1038/nmicrobiol.2016.195 27775707 PMC6872982

[B56] Prosser J. Head I. Stein L. (2014). “The family nitrosomonadaceae,” in , eds Dworkinm M. Falkow S. Rosenberg E. Schleifer K.-H. Stackebrandt E. (Berlin: Springer).

[B57] Purkhold U. Pommerening-röser A. Schmid M. C. Koops H.-P. Juretschko S. Wagner M. (2000). Phylogeny of all recognized species of ammonia oxidizers based on comparative 16S rRNA and amoA sequence analysis?: implications for molecular diversity surveys. 66 5368–5382. 10.1128/AEM.66.12.5368-5382.2000 11097916 PMC92470

[B58] R Development Core Team (2015). Available at: http://www.r-project.org

[B59] Reshetnikov A. S. Rozova O. N. Khmelenina V. N. Mustakhimov I. I. Beschastny A. P. Murrell J. C. (2008). Characterization of the pyrophosphate-dependent 6-phosphofructokinase from *Methylococcus capsulatus* Bath. 288 202–210. 10.1111/j.1574-6968.2008.01366.x 19054082

[B60] Santoro A. E. Dupont C. L. Richter R. A. Craig M. T. Carini P. McIlvin M. R. (2015). Genomic and proteomic characterization of “*Candidatus Nitrosopelagicus brevis*: an ammonia-oxidizing archaeon from the open ocean. 112 1173–1178. 10.1073/pnas.1416223112 25587132 PMC4313803

[B61] Sayavedra-Soto L. A. Hommes N. G. Russell S. A. Arp D. J. (1996). Induction of ammonia monooxygenase and hydroxylamine oxidoreductase mRNAs by ammonium in *Nitrosomonas europaea*. 20 541–548. 10.1046/j.1365-2958.1996.5391062.x 8736533

[B62] Schmidt I. Steenbakkers P. J. M. op den Camp H. J. M. Schmidt K. Jetten M. S. M. (2004). Physiologic and proteomic evidence for a role of nitric oxide in biofilm formation by *Nitrosomonas europaea* and other ammonia oxidizers. 186 2781–2788. 10.1128/JB.186.9.2781-2788.2004 15090520 PMC387797

[B63] Schramm A. De Beer D. Wagner M. Amann R. (1998). Identification and activities in situ of *Nitrosospira* and *Nitrospira* spp. as dominant populations in a nitrifying fluidized bed reactor. 64 3480–3485. 9726900 10.1128/aem.64.9.3480-3485.1998 PMC106750

[B64] Seaver L. C. Imlay J. A. (2001). Alkyl hydroperoxide reductase is the primary scavenger of endogenous hydrogen peroxide in *Escherichia coli*. 183 7173–7181. 10.1128/JB.183.24.7173-7181.2001 11717276 PMC95566

[B65] Sedlacek C. J. Nielsen S. Greis K. D. Haffey W. D. Revsbech P. Ticak T. (2016). Effects of bacterial community members on the proteome of the ammonia-oxidizing bacterium *Nitrosomonas* sp. Strain Is79. 82 4776–4788. 10.1128/AEM.01171-16 27235442 PMC4984276

[B66] Shively J. M. van Keulen G. Meijer W. G. (1998). Something from almost nothing: carbon dioxide fixation in chemoautotrophs. 52 191–230. 10.1146/annurev.micro.52.1.191 9891798

[B67] Sims G. K. Ellsworth T. R. Mulvaney R. L. (1995). Microscale determination of inorganic nitrogen in water and soil extracts. 26 303–316. 10.1080/00103629509369298

[B68] Speksnijder A. G. C. L. Kowalchuk G. A. Roest K. Laanbroek H. J. (1998). Recovery of a *Nitrosomonas*-like 16S rDNA sequence group from freshwater habitats. 21 321–330. 10.1016/S0723-2020(98)80040-4 9704117

[B69] Spivak M. Weston J. Bottou L. Käll L. Stafford W. (2009). Improvements to the percolator algorithm for peptide identification from shotgun proteomics data sets. 8 3737–3745. 10.1021/pr801109k 19385687 PMC2710313

[B70] Stein L. Y. (2011). “Heterotrophic nitrification and nitrifier denitrification,” in , eds Ward B. Arp D. Klotz M. (Washington, DC: ASM Press), 95–114. 10.1128/9781555817145.ch5

[B71] Stein L. Y. Arp D. J. Hyman M. R. (1997). Regulation of the synthesis and activity of ammonia monooxygenase in *Nitrosomonas europaea* by altering pH to affect NH3 availability. 63 4588–4592. 10.1128/aem.63.11.4588-4592.1997 PMC1389297 16535741

[B72] Stein L. Y. Campbell M. A. Klotz M. G. (2013). Energy-mediated vs. ammonium-regulated gene expression in the obligate ammonia-oxidizing bacterium, *Nitrosococcus oceani*. 4:277. 10.3389/fmicb.2013.00277 24062734 PMC3772326

[B73] Tappe W. Laverman A. Bohland M. Braster M. Rittershaus S. Groeneweg J. (1999). Maintenance energy demand and starvation recovery dynamics of *Nitrosomonas europaea* and *Nitrobacter winogradskyi* cultivated in a retentostat with complete biomass retention. 65 2471–2477. 10347029 10.1128/aem.65.6.2471-2477.1999 PMC91364

[B74] Tusher V. G. Tibshirani R. Chu G. (2001). Significance analysis of microarrays applied to the ionizing radiation response. 98 5116–5121. 10.1073/pnas.091062498 11309499 PMC33173

[B75] Tyanova S. Temu T. Sinitcyn P. Carlson A. Hein M. Y. Geiger T. (2016). The Perseus computational platform for comprehensive analysis of (prote)omics data. 13 731–740. 10.1038/nmeth.3901 27348712

[B76] Utåker J. B. Andersen K. Aakra A. Moen B. Nes I. F. (2002). Phylogeny and functional expression of ribulose 1,5-bisphosphate carboxylase/oxygenase from the autotrophic ammonia-oxidizing bacterium *Nitrosospira* sp. isolate 40KI. 184 468–478. 10.1128/JB.184.2.468-478.2002 11751824 PMC139566

[B77] Van Kessel M. A. H. J. Speth D. R. Albertsen M. Nielsen P. H. Op Den Camp H. J. M. Kartal B. (2015). Complete nitrification by a single microorganism. 528 555–559. 10.1038/nature16459 26610025 PMC4878690

[B78] Vizcaíno J. A. Csordas A. del-Toro N. Dianes J. A. Griss J. Lavidas I. (2016). 2016 update of the PRIDE database and its related tools. 44 D447–D456. 10.1093/nar/gkv1145 26527722 PMC4702828

[B79] Wang Y. Coleman-Derr D. Chen G. Gu Y. Q. (2015). OrthoVenn: a web server for genome wide comparison and annotation of orthologous clusters across multiple species. 43 W78–W84. 10.1093/nar/gkv487 25964301 PMC4489293

[B80] Wei X. Sayavedra-Soto L. A. Arp D. J. (2004). The transcription of the cbb operon in *Nitrosomonas europaea*. 150 1869–1879. 10.1099/mic.0.26785-0 15184573

[B81] Wei X. Yan T. Hommes N. G. Liu X. Wu L. McAlvin C. (2006). Transcript profiles of *Nitrosomonas europaea* during growth and upon deprivation of ammonia and carbonate. 257 76–83. 10.1111/j.1574-6968.2006.00152.x 16553835

[B82] Wells G. F. Park H. D. Yeung C. H. Eggleston B. Francis C. A. Criddle C. S. (2009). Ammonia-oxidizing communities in a highly aerated full-scale activated sludge bioreactor: betaproteobacterial dynamics and low relative abundance of Crenarchaea. 11 2310–2328. 10.1111/j.1462-2920.2009.01958.x 19515200

[B83] Wessels H. J. Gloerich J. van der Biezen E. Jetten M. S. Kartal B. (2011). , 1st Edn. New York City, NY: Elsevier Inc. 10.1016/B978-0-12-381294-0.00021-3

[B84] Whittaker M. Bergmann D. Arciero D. Hooper A. B. (2000). Electron transfer during the oxidation of ammonia by the chemolithotrophic bacterium *Nitrosomonas europaea*. 1459 346–355. 10.1016/S0005-2728(00)00171-7 11004450

[B85] Wijma H. J. Canters G. W. de Vries S. Verbeet M. P. (2004). Bidirectional catalysis by copper-containing nitrite reductase. 43 10467–10474. 10.1021/BI0496687 15301545

[B86] Wilhelm R. Abeliovich A. Nejidat A. (1998). Effect of long-term ammonia starvation on the oxidation of ammonia and hydroxylamine by *Nitrosomonas europaea*. 124 811–815. 10.1093/oxfordjournals.jbchem.a022184 9756628

[B87] Wisniewski J. R. Zougman A. Nagaraj N. Mann M. (2009). Universal sample preparation method for proteome analysis. 6 359–362. 10.1038/nmeth.1322 19377485

[B88] Wood N. J. Sørensen J. (2001). Catalase and superoxide dismutase activity in ammonia-oxidising bacteria. 38 53–58. 10.1016/S0168-6496(01)00173-8

[B89] Wrage N. Velthof G. L. Van Beusichem M. L. Oenema O. (2001). Role of nitrifier denitrification in the production of nitrous oxide. 33 1723–1732. 10.1016/S0038-0717(01)00096-7

[B90] Yoo J.-G. Bowien B. (1995). Analysis of the *cbbF* genes from *Alcaligenes eutrophus* that encode fructose-1,6-/sedoheptulose-1,7-bisphosphatase. 31 55–61. 10.1007/BF00294635 7767230

[B91] Yu R. Perez-Garcia O. Lu H. Chandran K. (2018). *Nitrosomonas europaea* adaptation to anoxic-oxic cycling: insights from transcription analysis, proteomics and metabolic network modeling. 615 1566–1573. 10.1016/j.scitotenv.2017.09.142 29055584

[B92] Zybailov B. Mosley A. L. Sardiu M. E. Coleman M. K. Florens L. Washburn M. P. (2006). Statistical analysis of membrane proteome expression changes in *Saccharomyces cerevisiae*. 5 2339–2347. 10.1021/pr060161n 16944946

