# Supplementary material for: Comparative Proteomics of Three Species of Ammonia-Oxidizing Bacteria
Source: Front Microbiol. 2018 May 14;9:938. doi: 10.3389/fmicb.2018.00938 (PMC5960693; doi:10.3389/fmicb.2018.00938)
Supplement: Supplementary file 4 [file Data_Sheet_1.pdf]

Supplementary Material

Comparative proteomics of three species of ammonia-oxidizing bacteria

Authors:
Jackie K. Zorz[1§], Jessica A. Kozlowski[2], Lisa Y. Stein[3], Marc Strous[1], Manuel Kleiner[4§]

[1] Department of Geoscience, University of Calgary, Calgary, AB, Canada, [2] Department of Ecogenomics and Systems Biology, University of Vienna, Vienna, Austria, [3] Department of Biological Sciences, University of Alberta, Edmonton, AB, Canada, [4] Department of Plant and Microbial Biology, North Carolina State University, Raleigh, NC, United States

§corresponding authors
Email address:
jacqueline.zorz@ucalgary.ca
manuel_kleiner@ncsu.edu

Supplementary Tables

Supplementary Table 1. Culture growth and proteomics data

	Parameter	*Nitrosomonas europaea*	*Nitrosospira multiformis*	*Nitrosomonas ureae*
**Culture Growth**	Average generation time during exponential growth in pre-cultures	0.61 ± 0.02 days	1.0 ± 0.2 days	1.3 ± 0.4 days
	Cell count in 6-11 day old pre-cultures before transfer	$1.32 \times 10^7 \pm 3.2 \times 10^6$ cells/mL	$2.2 \times 10^7 \pm 3.0 \times 10^6$ cells/mL	$1.94 \times 10^7 \pm 6.5 \times 10^6$ cells/mL
	Cell count in starved cultures at the end of the 24 hour experiment	$1.17 \times 10^7 \pm 4.3 \times 10^6$ cells/mL	$1.57 \times 10^7 \pm 4.9 \times 10^6$ cells/mL	$1.43 \times 10^7 \pm 4.2 \times 10^6$ cells/mL
	Ammonia concentration in in 6-11 day old pre-cultures before transfer	0.8 ± 1.3 mM	3.9 ± 1.6 mM	4.6 ± 1.6 mM
	Ammonia concentration in starved cultures after 24 hours of starvation	Not Detected	Not Detected	Not Detected
	Ammonia concentration in control cultures at the end of the 24 hour experiment	8.7 ± 0.6 mM	7.9 ± 0.4 mM	8.4 ± 0.3 mM
	Nitrite concentration in 6-11 day old pre-cultures before transfer	9.9 ± 1.0 mM	6.5 ± 1.3 mM	5.4 ± 1.5 mM
	Nitrite concentration in starved cultures after 24 hours of starvation	Not Detected	Not Detected	Not Detected
	Nitrite concentration in control cultures at the end of the 24 hour experiment	0.28 ± 0.1 mM	0.97 ± 0.1 mM	0.25 ± 0.1 mM
**Mass spectrometry**	MS/MS total	839518	914235	692064
	Average MS/MS per sample	139920	152373	138413
	PSMs	317610	396701	261843
	Peptide Groups	16528	20885	17394
	Proteins (all confidence)	2085	2457	2406
	Proteins (only high and medium confidence) (% of proteome expressed)	814 (32%)	1064 (37%)	891 (30%)
	Protein Groups	2046	2430	2339

Supplementary Table 2. Accession numbers of select proteins involved in ammonia oxidation and electron transfer included in Figure 1.

**Enzyme**	***N. europaea***	***N. multiformis***	***N. ureae***
Ammonia monooxygenase subunit A	Q04507	Q2YAW5	A0A0S3AG12
Ammonia monooxygenase subunit B	Q04508	Q2Y6K6	A0A0S3AFP0
Ammonia monooxygenase subunit C	H2VFV0<br>Q82T63<br>H2VFU7	Q2YAW6	A0A0S3AFN9<br>A0A0S3AI82
Hydroxylamine oxidoreductase	Q50925	Q2YA36<br>Q2YA35	A0A0S3AFC8
Nitrite reductase	Q82VX5	Q2Y7H8	A0A0S3AFD2
Nitric oxide reductase	Q82TA3	Q2Y9L3<br>Q2Y9L2	n/a
Nitrosocyanin	Q820S6	Q2Y8L9	A0A0S3AFA2
Cytochrome c-554	Q57142	Q2YA34	A0A0S3AN35
Cytochrome c-552	P95339	Q2YC69	A0A0S3AF59
Cytochrome P460 (cytL)	H2VFU9	n/a	A0A0S3AFC9
Cytochrome c' beta (cytS)	Q82W71	Q2Y648	A0A0S3AI53
Cytochrome cm-552	Q50926	Q2YA33	A0A0S3AK12
Cytochrome aa3	Q82VP9	Q2YCN0<br>Q2Y847	A0A0S3AH24
Cytochrome bc1	Q82VQ3	Q2Y849<br>Q2YCM6	A0A0S3AGL8

Supplementary Table 3. Accession numbers of enzymes involved in the CBB cycle for all three strains. Number in the first column corresponds to the numbers used in Figure 2b for enzyme identification

	**Enzyme**	***N. europaea***	***N. multiformis***	***N. ureae***
1	RubisCO large chain	Q82TG6	Q2YB78	A0A0S3AHX5<br>A0A0S3AFL1
1	RubisCO small chain	Q82TG7	Q2YB79	A0A0S3AHZ4<br>A0A0S3AFG4
2	Phosphoglycerate kinase	Q82XE8	Q2YC27	A0A0S3AI02
3	Glyceraldehyde-3-phosphate dehydrogenase	Q82XE7	Q2YC26	A0A0S3AHS1
4	Triosephosphate isomerase	Q82TU1	Q2YA29	A0A0S3AID8
5	Fructose-bisphosphate aldolase	Q82XF0	Q2Y8Q7<br>Q2YC29	A0A0S3AHR8<br>A0A0S3AN59
6	Sedoheptulose bisphosphatase	n/a	n/a	n/a
7	Fructose-1,6-bisphosphatase	Q82WY3	n/a	n/a
8	Transketolase	Q820H7<br>Q82XE6	Q2YBU5<br>Q2YC25	A0A0S3AHS4
9	Ribulose-phosphate-3-epimerase	Q82SZ5	Q2Y6G1	A0A0S3AGL5
10	Ribose-5-phosphate isomerase	Q82TX6	Q2YBX7	A0A0S3AHL4
11	Phosphoribulokinase	Q82UL0	Q2YBK1	A0A0S3AM16
12	Pyrophosphate-dependent phosphofructokinase	Q82TF4	Q2YB24	A0A0S3AI36
13	Inositol monophosphatase/type IV F1P6Pase	Q82TE3	Q2YB92	A0A0S3AHX4<br>A0A0S3AIS2

Supplementary Table 4. Description of ortholog information found in Supplementary excel tables 7-9

**Orthologous proteins found in the AOB genomes: *Nitrosomonas europaea* (Ne), *Nitrosospira multiformis* (Nm), and *Nitrosomonas ureae* (Nu)**		
All orthologs were identified using the program Orthovenn: http://www.bioinfogenome.net/OrthoVenn/		
**Table**	**Name**	**Description**
**7**	Single_gene_orthologs	Orthologs found in all three genomes but in only one copy per genome
**8**	Singletons	Expressed proteins from one genome that had no ortholog matches in the other two genomes
**9**	Two_species_orthologs	Orthologs found in 2 of three genomes in one copy per genome

Supplementary Table 5. Top 50 most abundant proteins (% relative abundance) for each species investigated in this study. If genes are orthologous between species they are displayed in the same row.

N. europaea Accession	*N. multiformis* Accession	*N. ureae* Accession	Description	*N. europaea*	*N. multiformis*	*N. ureae*
Q82Y61	Q2Y6I5	A0A0S3AG00	10 kDa chaperonin	0.846225	1.574932	1.86374
Q82X89	Q2YAZ8	A0A0S3AJF2	30S ribosomal protein S10	0.676294	0.323518	0.470631
Q82XS9	Q2Y5X8	A0A0S3ALZ6	30S ribosomal protein S15	0.451646	0.290156	0.308619
Q82TZ6	Q2YBA7	A0A0S3AH93	30S ribosomal protein S2	0.522609	0.385369	0.517171
Q82XN1	Q2Y7B7	A0A0S3AI43	30S ribosomal protein S21	0.610866	0.283116	0.293264
Q82X70	Q2YAX3	A0A0S3AJF4	30S ribosomal protein S4 A	0.437628	0.245854	0.351479
Q82X77	Q2YAY3	A0A0S3AJR2	30S ribosomal protein S8	0.12818	0.437497	0.21617
Q82T72	Q2YB08	A0A0S3AKH3	50S ribosomal protein L1	0.429505	0.364462	0.722339
Q82U36	Q2YBJ1	A0A0S3AG28	50S ribosomal protein L19	0.891615	0.474541	0.635273
Q820Q9	Q2YAY5	A0A0S3AJC4	50S ribosomal protein L5	0.457054	0.335299	0.400319
Q82T74	Q2YB06	A0A0S3AKV6	50S ribosomal protein L7/L12	0.814855	0.408266	0.599014
Q82XQ9	Q2Y7M8	A0A0S3AGH1	50S ribosomal protein L9	0.446776	0.457042	0.342599
Q82Y60	Q2Y6I6	A0A0S3AFK1	60 kDa chaperonin	1.691542	1.393075	1.874853
Q82U57	Q2YA43	A0A0S3AGN4	Acyl carrier protein	0.596629	0.60277	0.352774
Q820H3			Alkyl hydroperoxide reductase	0.913294	0	0
Q04507	Q2YAW5	A0A0S3AG12	Ammonia monooxygenase subunit A	1.278051	0.879039	0.614642
Q04508	Q2Y6K6	A0A0S3AFP0	Ammonia monooxygenase subunit B	3.456044	3.806341	3.490765
H2VFU7	Q2YAW6	A0A0S3AI82/ A0A0S3AFN9	Ammonia monooxygenase subunit C	0.434124	0.223191	0.288361
Q82XQ0	Q2YCA5	A0A0S3AHF6	ATP synthase subunit alpha	1.107305	0.592557	0.88902
Q82XQ2	Q2YCA7	A0A0S3AGP9	ATP synthase subunit b	0.554859	0.19427	0.672047
Q82XP8	Q2YCA3	A0A0S3AH35	ATP synthase subunit beta	1.135587	0.700324	0.984254
Q82SU7	Q2Y6J3	A0A0S3AK75	Bacterial histone-like DNA-binding protein	1.768732	0.851644	1.095308
Q82XN8	Q2Y5H1	A0A0S3AGT0	Bacterial outer membrane protein 1	1.791239	0.62275	1.467036
Q82S16	Q2Y6Z1	A0A0S3AMB4	Bacterial outer membrane protein 2	1.598599	1.349047	0.439786
Q82W35	Q2Y6D6	A0A0S3AG51	Bacterioferritin 1	0.244802	0.514984	0.017083
		A0A0S3AHF1	Bacterioferritin 2	0	0	0.517586
O06430	Q2Y6U0	A0A0S3AMV5	Chaperone protein DnaK	0.312235	0.53606	0.48511
Q82TY4	Q2Y6T3	A0A0S3AGH0	Cold-shock DNA-binding domain	0.485136	0.413581	0.162724
Q82T65	Q2Y5C3	A0A0S3AIQ1	Copper resistance protein CopC	0.210375	0.793218	0.094398
	Q2Y9S4		CsbD-like protein	0	0.447554	0
Q82WJ0	Q2YCN0	A0A0S3AH24	Cytochrome c oxidase subunit 2	0.502984	0.940563	1.033047
Q820P5	Q2Y5I3	A0A0S3AHP2	Cytochrome c, class I (1)	0.29482	0.857655	1.147102
Q82Y64	Q2YAF6	A0A0S3AI05	Cytochrome c, class I (2)	0.698381	0.483805	0.485143
Q820P4	Q2Y5I2	A0A0S3AHN4	Cytochrome c, class I (3)	0	0.398543	0.035166
P55929	Q2YBW5	A0A0S3AK60	Cytochrome c551 peroxidase	0.593571	0.300997	0.676175
P95339	Q2YC69	A0A0S3AF59	Cytochrome c-552	1.656558	0.544832	0.975231

N. europaea Accession	*N. multiformis* Accession	*N. ureae* Accession	Description	*N. europaea*	*N. multiformis*	*N. ureae*
Q57142	Q2YA34	A0A0S3AN35	Cytochrome c-554	0.38086	0.567094	0.324274
Q81ZS3	Q2YAZ9	A0A0S3AJP6	Elongation factor Tu	1.288032	0.956967	0.969499
O85348	Q2Y9P0	A0A0S3AMG9	Enolase	0.206378	0.405045	0.17379
		A0A0S3AMA6	Entry exclusion lipoprotein TrbK	0	0	0.88014
Q82UA3	Q2Y9D1	A0A0S3AJU9	Flagellin	0	0.425687	0.005116
Q82XF0	Q2YC29	A0A0S3AHR8	Fructose-bisphosphate aldolase, class-II	0.615506	0.573238	0.518293
Q82S02	Q2Y5X1	A0A0S3AG80	General diffusion Gram-negative porins	4.362663	1.130986	1.975695
Q82XE7	Q2YC26	A0A0S3AHS1	Glyceraldehyde-3-phosphate dehydrogenase	0.528227	0.466577	0.398451
Q82T55	Q2Y862	A0A0S3AHM5	Heat-shock protein Hsp20	0.110254	0.018025	0.912376
Q50925	Q2YA36	A0A0S3AFC8	Hydroxylamine oxidoreductase	0.813608	0.56955	0.734522
	Q2YBA8		Membrane-anchored ribosome-binding protein	0	0.839309	0
Q82S91			Metal-binding protein SmbP	0.826463	0	0
		A0A0S3AFD2	Nitrite reductase	0	0	2.88794
Q820S6	Q2Y8L9	A0A0S3AFA2	Nitrosocyanin	1.690333	1.803488	2.270346
Q82UR3	Q2Y764	A0A0S3AG66	Peptidylprolyl isomerase	0.949204	0.904461	0.904942
		A0A0S3AN75	Peroxidase	0	0	0.519497
Q82UL0	Q2YBK1	A0A0S3AM16	Phosphoribulokinase	0.637692	0.218003	0.269243
	Q2YCH1		Polysaccharide export outer membrane protein	0	0.507212	0
	Q2YCK2	A0A0S3AKS4	Probable thiol peroxidase	0	0.633906	0.63237
Q82V25	Q2YBW8	A0A0S3AN41	Protease FtsH subunit HflK	0.494951	0.223529	0.458692
Q82V26	Q2YBW9	A0A0S3AK31	Protein HflC	0.657266	0.226138	0.784248
Q82XZ4	Q2Y859	A0A0S3ALY3	Putative exosortase-associated protein	0.097042	0.416571	0.093435
Q82XW6			Putative lipoprotein	0.637878	0	0
Q82T08			Putative peptidoglycan binding domain 1	0.523396	0	0
Q82TG6		A0A0S3AFL1	RuBisCO Large Form Iaq	1.10913	0	0.518812
	Q2YB78	A0A0S3AHX5	RuBisCO Large Form IC	0	1.788213	0.81335
Q82TG7		A0A0S3AFG4	RuBisCO Small Form Iaq	0.505822	0	0.17916
	Q2YB79	A0A0S3AHZ4	RuBisCO Small Form IC	0	2.375144	0.942758
	Q2Y7X9	A0A0S3AFT3	Rubrerythrin	0	1.226076	1.222623
Q82W28	Q2YBR2	A0A0S3ALT7	Superoxide dismutase	0.193307	0.450574	0.102243
Q82VN2	Q2Y793	A0A0S3AKZ2	Thioredoxin	0.631354	0.744102	0.631109
		A0A0S3AKX1	Transcriptional regulator	0	0	0.686909
Q82U33	Q2Y5K7	A0A0S3AGF6	Uncharacterized protein 1	0.227662	0.672991	0.442144
Q82V11		A0A0S3AFG5	Uncharacterized protein 10	0.604306	0	0.654737
Q82T66	Q2Y5C5		Uncharacterized protein 11	0.675596	0.409756	0
	Q2YCH2		Uncharacterized protein 12	0	0.515004	0
		A0A0S3AIE3	Uncharacterized protein 13	0	0	0.498152
		A0A0S3AI57	Uncharacterized protein 14	0	0	0.778513
Q82VW3	Q2Y5C2		Uncharacterized protein 15	0.977299	0.427238	0

N. europaea Accession	*N. multiformis* Accession	*N. ureae* Accession	Description	*N. europaea*	*N. multiformis*	*N. ureae*
		A0A0S3AMF4	Uncharacterized protein 16	0	0	0.452306
Q82TN1			Uncharacterized protein 17	0.986341	0	0
Q82VB7	Q2Y6Q8	A0A0S3AGF9	Uncharacterized protein 2	0.395486	0.140708	0.470865
Q82VY9			Uncharacterized protein 3	0.754294	0	0
	Q2Y9X6		Uncharacterized protein 4	0	0.807647	0
	Q2Y9X8		Uncharacterized protein 5	0	0.687904	0
		A0A0S3AFI8	Uncharacterized protein 6	0	0	0.78225
		A0A0S3AIB4	Uncharacterized protein 7	0	0	1.54947
		A0A0S3AKF1	Uncharacterized protein 8	0	0	0.713927
Q82TI0			Uncharacterized protein 9	2.156594	0	0

* Proteins without accession numbers do not contain an ortholog for that gene.

Supplementary Table 6. Spearman correlation values between generation time of cultures and protein expression. The final column specifies if the protein is expressed to a higher level in faster or slower growing cells. All relationships are significant $p < 0.05$

N. europaea **Accession**	***N. multiformis*** **Accession**	***N. ureae*** **Accession**	**Protein**	**Spearman Correlation**	
Q82XJ5	Q2Y5Q2	A0A0S3AGP1	Methionine aminopeptidase	-0.93	Faster
Q82UK0	Q2YBK2	A0A0S3AIX5	50S ribosomal protein L13	-0.93	Faster
Q82U38	Q2YBJ4	A0A0S3AGI4	30S ribosomal protein S16	-0.93	Faster
Q82X87	Q2YAZ6	A0A0S3AJE3	50S ribosomal protein L4	-0.92	Faster
Q82XE6	Q2YC25	A0A0S3AHS4	Transketolase	-0.9	Faster
Q82UH7	Q2YAV7	A0A0S3AH46	2-alkenal reductase	-0.88	Faster
Q820R9	Q2Y6Y7	A0A0S3AJB3	Prephenate dehydratase	0.88	Slower
Q82X83	Q2YAZ2	A0A0S3AJB6	50S ribosomal protein L22	-0.88	Faster
Q82Y61	Q2Y6I5	A0A0S3AG00	10 kDa chaperonin	0.88	Slower
Q82VV2	Q2YBS5	A0A0S3AMP6	Translation initiation factor IF-3	0.87	Slower
Q82TV8	Q2YA09	A0A0S3AIF6	Chaperone protein HtpG	0.87	Slower
Q82WB8	Q2Y7A2	A0A0S3AMD6	Dihydrolipoamide dehydrogenase	0.87	Slower
Q82X86	Q2YAZ5	A0A0S3AJE4	50S ribosomal protein L23	-0.87	Faster
Q82XQ6	Q2Y7M5	A0A0S3AGW2	30S ribosomal protein S6	-0.87	Faster
Q82U47	Q2YBT3	A0A0S3AMI5	Transcription elongation factor GreA	0.87	Slower
Q82VT2	Q2Y629	A0A0S3AHI1	Transcriptional regulator MraZ	0.85	Slower
Q82VZ3	Q2Y826	A0A0S3AFK9	ATP dependent zinc metalloprotease FtsH	-0.85	Faster
Q82UR0	Q2YA84	A0A0S3AME4	Protease HtpX homolog	-0.85	Faster
Q82TL0	Q2YCS7	A0A0S3AGB8	Oxygen dependent coproporphyrinogen III oxidase	-0.85	Faster
Q82TR4	Q2YA39	A0A0S3AGX9	Succinate CoA ligase ADP forming subunit beta	-0.85	Faster
Q82WI5	Q2Y7Q9	A0A0S3AFU9	Aspartate semialdehyde dehydrogenase	0.85	Slower
Q82VE9	Q2Y6A4	A0A0S3AJ65	Preprotein translocase subunit YajC	0.85	Slower
Q82S95	Q2Y798	A0A0S3AJD6	Delta aminolevulinic acid dehydratase	-0.83	Faster
Q82VA9	Q2YBL2	A0A0S3AHB1	Glycine tRNA ligase alpha subunit	-0.83	Faster
Q820P5	Q2Y5I3	A0A0S3AHP2	Cytochrome C	0.83	Slower
Q82SM8	Q2YBB8	A0A0S3AJ95	Cell division protein ZipA	-0.83	Faster
Q82VQ4	Q2YCM4	A0A0S3AH18	SURF1 like protein	0.82	Slower
Q82W40	Q2Y9W8	A0A0S3AGT7	Pyridine nucleotide disulfide oxidoreductase	0.82	Slower
Q82TU6	Q2YA24	A0A0S3AIH8	NADH quinone oxidoreductase subunit D	-0.82	Faster
Q82TU9	Q2YA21	A0A0S3AIH4	NADH quinone oxidoreductase	-0.82	Faster
Q82X88	Q2YAZ7	A0A0S3AJB1	50S ribosomal protein L3	-0.82	Faster
Q82Y48	Q2Y6P2	A0A0S3AHI6	Rhodanese	0.8	Slower

N. europaea Accession	*N. multiformis* Accession	*N. ureae* Accession	Protein	Spearman Correlation	
Q82W16	Q2YBP4	A0A0S3AIW7	4-hydroxythreonine-4-phosphate dehydrogenase	-0.8	Faster
Q82XT2	Q2YB74	A0A0S3AHL0	Transporter 2	-0.8	Faster
Q82UL0	Q2YBK1	A0A0S3AM16	Phosphoribulokinase	-0.8	Faster
Q82XC9	Q2YA70	A0A0S3AKR6	Biopolymer transporter ExbB	-0.8	Faster
Q82TZ7	Q2YBA5	A0A0S3AHB8	Uridylate kinase	0.8	Slower
Q82X82	Q2YAZ0	A0A0S3AJA4	50S ribosomal protein L16	-0.8	Faster
Q81ZS3	Q2YAZ9	A0A0S3AJP6	Elongation factor Tu	-0.8	Faster

Supplementary tables 7-9 are available as separate files and contain statistical information about orthologous proteins identified in this study. Supplementary table 4 contains information on the contents of these files.

Supplementary Figures

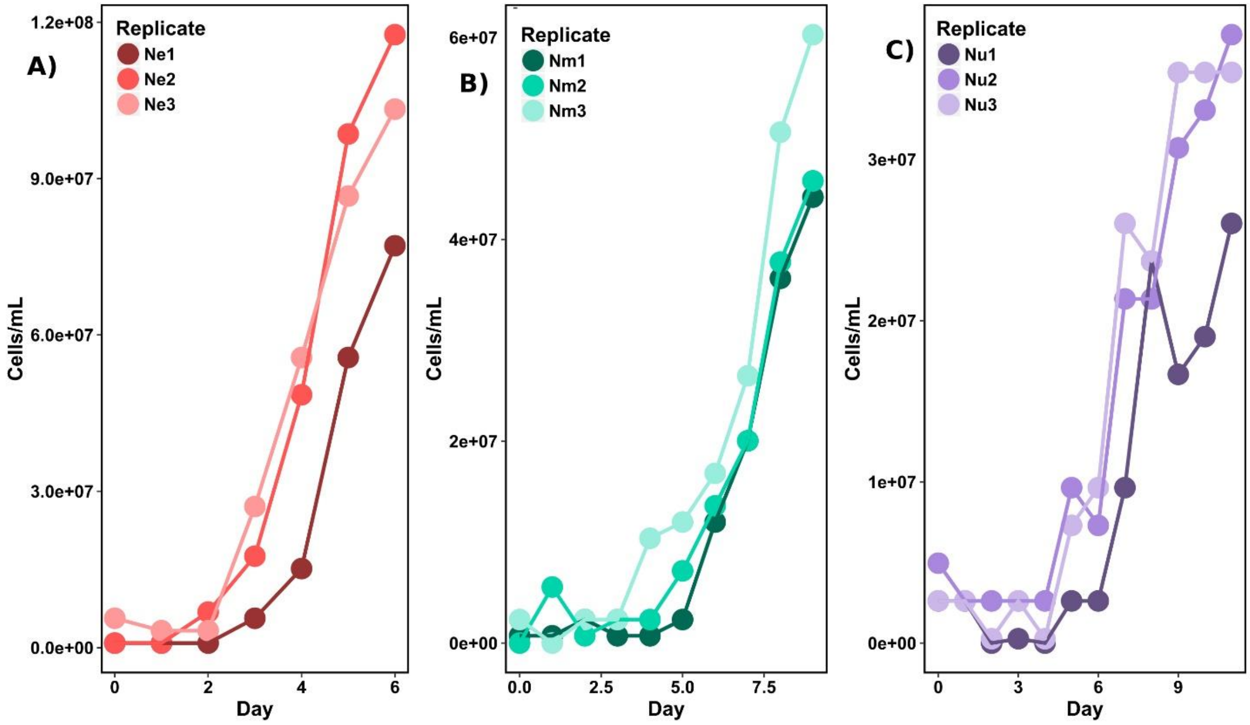

Supplementary Figure 1. Growth curves of the triplicate cultures, which were used for the starvation experiment. Cultures were spun down for the starvation experiment on the last day of cell density measurements. A) *N. europaea*, B) *N. multiformis*, C) *N. ureae*. Note that y-axis scales are not comparable. Average generation time during exponential growth for *N. europaea*: 0.61 ± 0.02 days, *N. multiformis*: 1.0 ± 0.2 days, *N. ureae*: 1.3 ± 0.4 days.

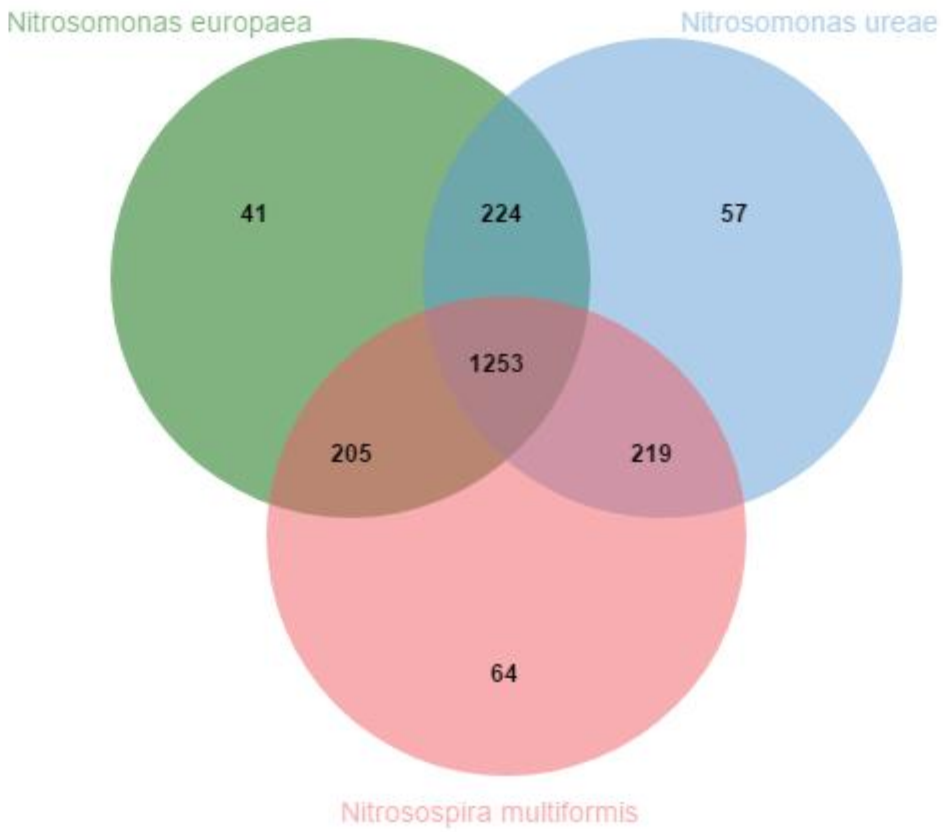

Supplementary Figure 2. Venn Diagram of paralogous and orthologous genes found in *N. europaea*, *N. ureae*, and *N. multiformis* produced by Orthovenn. Not included in this diagram are the singleton genes which have no orthologs or paralogs in these three species.

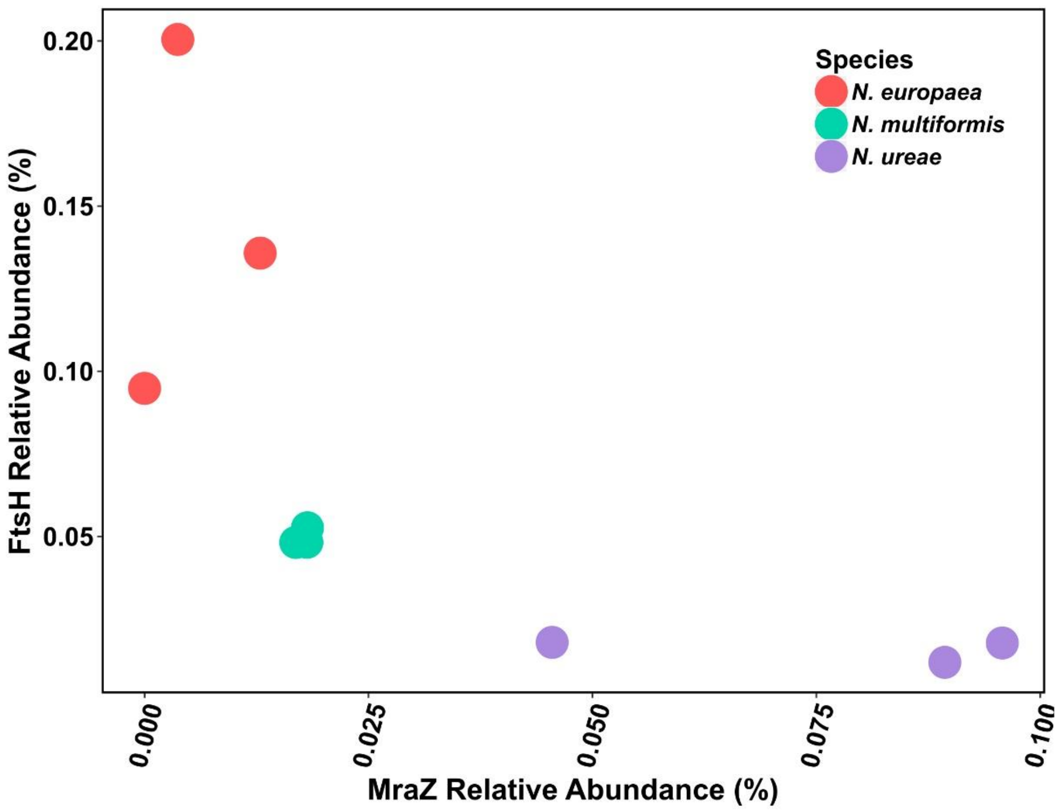

Supplementary Figure 3. Negative trend between MraZ relative abundance and FtsH relative abundance. Spearman correlation = -0.87, $p < 0.005$.
